# Modeling Hemorrhagic Shock in Male SD Rats by Fixed Volume Blood Withdrawal Followed by Partial Resuscitation and Long-Term Outcome Assessment

**DOI:** 10.3390/medsci14050587

**Published:** 2026-09-18

**Authors:** Alina M. Ismailova, Victor A. Palikov, Maria S. Severyukhina, Elena A. Tukhovskaya, Arina V. Kholina, Svetlana G. Semushina, Gulsara A. Slashcheva, Elvira R. Shaykhutdiniva, Irina N. Kravchenko, Vitaly A. Kazakov, Ekaterina N. Kazakova, Marina S. Kazakova, Alena B. Timakina, Maksim V. Shinelev, Vladimir A. Rykov, Olga I. Patsap, Igor A. Dyachenko, Arkady N. Murashev

**Affiliations:** 1 Branch of Shemyakin and Ovchinnicov Institute of Bioorganic Chemistry, Russian Academy of Sciences, Prospekt Nauki, 6, Pushchino 142290, Russia; 2 Pushchino Branch of the Federal State Budgetary Educational Institution of Higher Education, Russian Biotechnological University (BIOTECH University), Pushchino 142290, Russia; 3 Research and Educational Resource Center for Immunophenotyping, Digital Spatial Profiling and Ultrastructural Analysis Innovative Technologies (Molecular Morphology Center), Peoples’ Friendship University of Russia Named After Patrice Lumumba, Miklukho-Maklaya Street, 6, Moscow 117198, Russia

**Keywords:** blood loss, hemorrhagic shock, rats, preclinical studies, animal models, resuscitation

## Abstract

**Background/Objectives**: Hemorrhagic shock (HS) is one of the leading causes of disability and mortality in intensive care units. Animal modeling of HS is important for preclinical research to elucidate mechanisms and develop treatments. **Methods**: Adult male SD rats were divided into three groups, half of which were euthanized on day 8 and the other half on day 29 after HS. HS was simulated in awake animals by withdrawing blood from the carotid artery at a volume of 40% of circulating blood volume (CBV). One hour after HS, volume resuscitation therapy (VRT) was made by administering albumin-containing saline (AS) or autologous blood (AB) at 20% of CBV. Arterial pressure (AP), heart rate (HR), body temperature, blood gases, and blood metabolites, body weight gain, hemostasis, and blood cellular composition were analyzed. On days 8 and 29, bone marrow composition, organ weight, and histology were analyzed. **Results**: During blood withdrawal, AP dropped, HR increased, and hypothermia developed. After HS and subsequent VRT, 30% of animals receiving SA died, while all animals receiving AB survived. Following HS, a negative weight gain trend, anemia, pronounced inflammatory reaction, and coagulopathy developed. After HS, animals experienced an increase in partial oxygen pressure, a decrease in partial carbon dioxide pressure, total arterial oxygen content in arterial blood, stress-induced glycemia, and metabolic lactatemia. In bone marrow, the number of erythroid cells increased, while leukocyte and lymphocyte numbers decreased. HS resulted in increased spleen weight with increased extramedullary hematopoiesis. Ischemic kidney tissue damage with a decrease in kidney weight was observed, as well as post-ischemic accidental thymus involution with a catastrophic decrease in its weight. In addition, ischemic brain damage was observed in some animals. Animals showed a compensatory increase in hematopoiesis. All changes were less pronounced in AB-treated animals. **Conclusions**: We have developed a suitable model of HS, comparable to a similar condition in humans. This model can be easily reproduced and applied in most laboratories conducting preclinical studies.

## 1. Introduction

Massive blood loss accompanied by HS remains one of the leading causes of mortality in intensive care, acting as a key pathogenetic link in the outcome of a wide range of emergency conditions [1]. Thus, HS is the leading cause of death in trauma patients worldwide [2,3], and the highest probability of a fatal outcome is observed in the first hours after injury at the prehospital stage [4,5]. In patients with injuries of various origins, HS is the cause of death—in 30% of cases in patients with complications of gastrointestinal diseases, in 60% of cases—in patients with abdominal aortic aneurysm—in 100% of cases, and in obstetric and gynecological complications—in 23% of cases [6].

The pathophysiological mechanisms of HS development are associated with acute hypovolemia and, consequently, impaired peripheral perfusion and a critical reduction in oxygen delivery. Acute hypovolemia results in a compensatory release of vasopressin, vasoconstriction to maintain blood pressure and cardiac output, and an increase in heart rate. Compensatory centralization of blood circulation occurs, meaning that the bulk of the blood is redirected to the organs most sensitive to hypoxia—the brain and heart—due to increased venous outflow due to venous spasm and the emptying of hematopoietic depots (spleen, liver, skin, adipose, and muscle capillaries). Physiological compensation for blood loss also includes autohemodilution (fluid entering the bloodstream from the interstitial space), activation of hematopoiesis, and hypercoagulation.

A decrease in oxygen supply leads to a transition from an aerobic to an anaerobic metabolic pathway, which causes the accumulation of oxygen radicals, lactate, and the development of acidosis [7], as well as a decrease in body temperature [8]. Anaerobic metabolism leads to massive mitochondrial dysfunction, ATP depletion, and cell death [5,9]. With prolonged uncompensated HS, persistent hypoperfusion, hypotension, hypoxia, disseminated intravascular coagulation, and multiple organ failure develop [10,11,12]. In addition, the immune system responds to acute blood loss by activating innate immune genes responsible for pro- and anti-inflammatory processes [13,14]. In patients with severe uncompensated bleeding, inflammation persists, leading to systemic inflammatory response syndrome, multiple organ failure, and death [15]. Moreover, if the hyperinflammatory response is activated early in the blood loss process, immunosuppression subsequently develops, which can lead to long-term complications and death [16]. Although severe bleeding is life-threatening, it can be treated in a hospital setting if properly diagnosed and treated promptly [4,17,18,19]. Time is a key factor in the effectiveness of therapy in acute severe blood loss; that is, the sooner therapy is started, the higher the chances of its success [5,20,21], and it is important to minimize the duration of the pre-hospital stage of emergency care [22]. The primary goal of HS therapy is to stop bleeding and maintain organ and tissue perfusion and oxygenation, i.e., volume resuscitation therapy (VRT). However, proper VRT presents a significant therapeutic challenge, as aggressive intravenous fluid administration can lead to dilution of coagulation factors. Therefore, VRT aims to maintain “acceptable” hypotension and tissue perfusion. However, this strategy should be used with caution in patients with concomitant hypertension (elderly patients) and in patients with traumatic brain injury (it is necessary to maintain normal perfusion pressure to ensure adequate oxygenation) [23,24,25]. The use of 0.9% saline may increase acidosis and kidney injury in critically ill patients, in contrast to isotonic balanced crystalloid solutions containing physiological concentrations of electrolytes, although the advisability of their use in general is questionable [26,27,28].

Currently, emergency care includes transfusion of equal volumes of fresh frozen plasma, platelets, and red blood cells in a ratio of 1:1:1 or 1:1:2 [25,29]. Whole blood transfusion is considered in some studies as a more effective strategy for maintaining homeostasis [30,31]. Assessing the overall condition and the specific phase of HS is crucial for choosing a treatment strategy and predicting outcome, as certain therapeutic actions during one phase may be harmful during another. The interaction between inflammation, hypoxia, and coagulation during the development of HS constantly alters the pathophysiological balance, requiring thoughtful and precise interventions based on clinical and laboratory diagnostic results [32]. Important evaluation and prognostic parameters are AP and HR, measurement of blood gases and metabolites, measurement of lactate levels, hemostasis parameters, and hematological analysis of whole blood [33,34,35,36,37]. Animal models of HS are used to study the pathophysiology of HS and research new therapeutic strategies for its correction. HS is most often modeled in anesthetized animals, with blood drawn until blood pressure drops to a certain level (35–45 mmHg) [38,39]. However, when HS is modeled by fixed blood pressure, the amount of blood removed is not uniform, as compensatory mechanisms may be activated at different times and to different degrees in different animals. Furthermore, anesthesia eliminates the neurogenic factor and activates the parasympathetic nervous system, masking the body’s sympathetic responses. In our opinion, modeling HS based on the volume of blood loss relative to the CBV is more relevant [40,41]. It should be noted that the vast majority of studies using HS modeling consider short-term modeling effects over periods of 3 to 72 h [42,43,44,45].

The aim of our study was to develop an optimal blood loss model of HS in awake male SD rats that would be reproducible in most preclinical laboratories. We also planned to examine the long-term effects of HS using multiple time points to record parameters, starting from baseline (BL) and up to day 29 post-HS.

## 2. Materials and Methods

### 2.1. Animals

The study used sexually mature male SD rats, specific pathogen-free (SPF), aged 12–14 weeks with an average body weight of 327 ± 17 g. The animals were obtained from the Pushchino Laboratory Animal Breeding Center in Pushchino, Russia. Upon receipt, the animals were quarantined for 14 days. Clinically healthy animals were included in the study. The animals’ clinical health was confirmed by examining them for any abnormalities in appearance and behavior. Animals were randomly assigned to groups using body weight as a criterion. It was ensured that the average body weight did not differ between groups, and that the variation in body weight within groups did not exceed 20%. During the study, the animals were kept under controlled environmental conditions in a barrier zone with a “clean” and “dirty” corridor system with controlled environmental conditions (temperature 20–24 °C, relative humidity 30–55%, 12 h light cycle; 08:00–20:00—“day”, 20:00–08:00—“night”; 10-fold change in air volume in the room per hour). The animals received ad libitum food: laboratory mice and rats received Velaz FORTI 1324 Maintenance Diet (Altromin Spezialfutter GmbH & Co KG, Lage, Germany). All procedures and manipulations with animals were approved by the Committee for Control over Care and Use of Laboratory Animals of BIBCh RAS (IACUC) (protocol number 1053/25 from 17 October 2025) and were carried out in accordance with the EU Directive 2010/63/EU. The actual start date for work with animals is 3 November 2025. The animal study was supported by the Scientific Infrastructure Project 075-15-2025-514/MSHE RF.

### 2.2. Design Description

The study involved 30 animals divided into three groups of 10 animals each, half of which were euthanized on day 8 and the other half on day 29 (Table 1). Animals from groups 1 and 2 had catheters implanted in the jugular vein to perform VRT and in the carotid artery to record AP and HR. HS was modeled by withdrawing blood in a volume of 40% of the CBV. One hour after HS modeling, partial VRT was performed by administering SA (Saline + Albumin (7:1)) or AB through a venous catheter in a volume equal to 50% of the withdrawn volume (i.e., 20% of the CBV). The CBV was calculated as 64 mL/kg [46,47]. Forty-eight hours after the HS modeling, the animals were again anesthetized with inhalation anesthesia to remove the catheters from the carotid artery and jugular vein. Animals from group 3 were intact, that is, they had not undergone any procedures during their lives.

Animals’ blood pressure and heart rate were recorded throughout the blood loss period and at 1 h, 3 h, 24 h, and 48 h after the simulated HS. Body temperature was measured before the start of HS, before VRT (60 min after the start of HS), 1 h, 3 h, 24 h, and 48 h after VRT. Oximetry parameters were measured in arterial blood samples taken from animals before HS modeling, 1 h after HS before the start of VRT, and 3, 24, and 48 h after HS modeling. Hematological parameters were measured in whole blood samples collected from animals before HS, 1 h after HS before the start of VRT, 3 h, 24 h, 48 h, and on days 8 and 29 after HS. Hemostasis parameters were measured in blood samples collected before HS, 24 h, 48 h, and on days 8 and 29 after HS.

During the intravital phase of the study, the condition of the animals was monitored, and body weight gain was recorded. At the end of the intravital phase of the study (8 days or 29 days), the animals were euthanized under anesthesia and total blood sampling was performed from the inferior vena cava; organs were weighed, and bone marrow smears were prepared for myelogram analysis.

**Figure 1:**
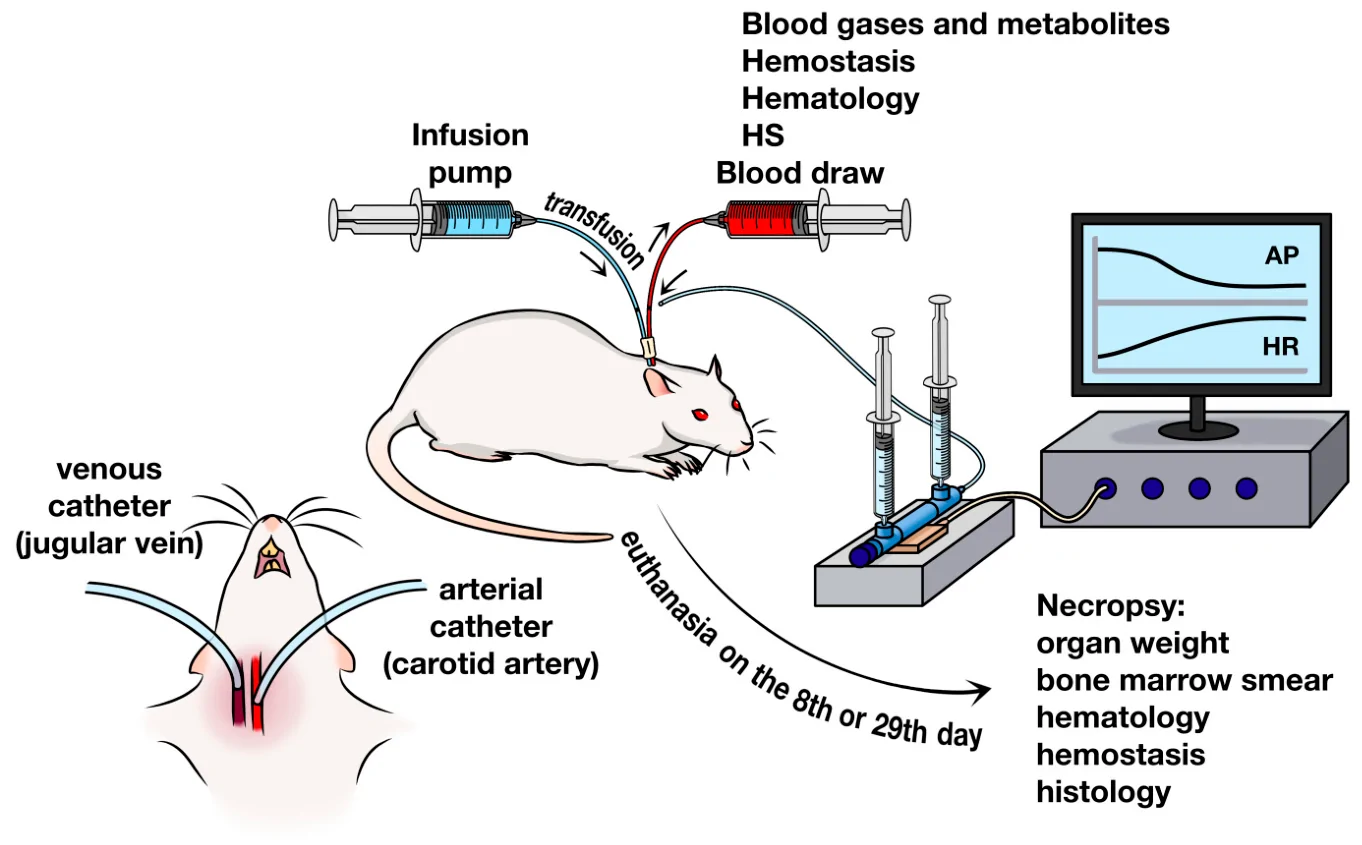
Experimental scheme.

Table 1 provides a description of animal groups, procedures performed, and drugs used, and Figure 1 shows experimental design.

### 2.3. Drugs in the Study

The study used 0.9% sodium chloride infusion solution (Solopharm, Saint Petersburg, Russia) and 20% human albumin infusion solution (Microgen, Moscow, Russia). Isoflurane inhalation anesthesia solution (Laboratories Karizoo, Barcelona, Spain) was used for anesthesia for HS modeling and subsequent catheter removal. For anesthesia for euthanasia, Vezotil^®^ (Moscow Endocrine Plant, Federal State Unitary Enterprise, Moscow, Russia) and Xyla^®^ injection solution (Interchemie, Venray, The Netherlands) were used. To prepare a preservative/anticoagulant for autologous blood, we used sodium citrate dihydrate (Servisbio, Wuhan, China), citric acid anhydrous extrapure (Sisco Research Laboratories PTV. LTD, Mumbai, India), dextrose monohydrate extrapure AR, ACS, ExiPlus, Multi-Compendial (Sisco Research Laboratories PTV. LTD, Mumbai, India), and water for injection (Solopharm, Saint Petersburg, Russia). Enroflon^®^ 2.5% antibiotic for injection (VIC-Animal Health, Vitebsk, Belarus) was used to prevent infection.

### 2.4. HS Modeling and VRT

To simulate HS, animals underwent catheterization of the external carotid artery (for blood sampling, AP, and HR measurements) and the jugular vein (for VRT). Animals were anesthetized with isoflurane (2.5% at a flow rate of 1.5 L/min), and the surgical field in the ventral and dorsal regions of the neck was prepared: hair was removed, and the skin was treated with a disinfectant solution. Animals were immobilized by tying forepaws and upper incisors to the operating table with soft ropes. During surgery, animals lay on a thermostatically controlled surface at a temperature of 37 °C. The surgery was performed using sterile instruments and materials, observing aseptic technique.

To implant a catheter into the carotid artery, a longitudinal skin incision 1.5–2.0 cm long was made along the midline of the neck. The salivary glands and muscles were carefully retracted. A section of the common trunk of the carotid artery approximately 0.7 cm long was carefully isolated. Ligatures were placed under the isolated section of the artery. A longitudinal incision 0.5 mm long was made in the vessel between the ligatures using vascular scissors. The tip of the catheter was inserted into the resulting opening. After releasing the distal ligature, the catheter was pushed further along the vessel to the shock-absorbing ring (by 2–3 cm). The ligatures were tightened and tied. Implantation of the catheter into the jugular vein was performed similarly to insertion of a catheter into the carotid artery. To expose the ends of the catheters to the withers, a longitudinal skin incision no longer than 5 mm was made on the back of the neck. Using a metal atraumatic tube, the catheter ends were subcutaneously advanced to the withers. The skin incision on the midline of the neck and the withers was closed with sterile absorbable suture material (Caproag 4/0, Repromed, Moscow, Russia). Following catheterization, animals were allowed to recover from anesthesia for 30 min (during this period, the animal’s vegetative status is completely restored), after which AP and HR were recorded for 5 min. Blood was then withdrawn from the arterial catheter in a bolus for oximetry (75 μL), hematology (200 μL), and hemostasis (0.5 mL), for a total volume of 775 μL. To simulate HS, the remaining blood was gradually withdrawn (so that the final volume of blood collected was 40% of the CBV), with equal volumes being withdrawn every 30 s for 15 min. CBV was calculated for each animal based on its body weight. During blood withdrawal for HS, external manifestations of stress in behavior and HR were monitored. Upon completion of blood collection, animals were left in a holding cage for 1 h, after which VRT in a volume of 20% of CBV was performed. To prevent infection, animals were administered the antibiotic Enroflon intramuscularly at a dose of 5 mg/kg and a volume of 2 mL/kg for 5 days after HS. To prevent blood clots, the catheters were filled with sterile saline and flushed with 100–150 μL of saline every 12 h for 48 h until the catheters were removed.

### 2.5. VRT Procedure

Animals in group 1 were given 0.9% sodium chloride solution (saline) followed by 20% albumin (7:1 *v*/*v*). The introduction of albumin should improve the maintenance of oncotic pressure [48]. Animals from group 2 were administered AB, which was chosen as TF therapy as the most optimal VRT option in case of blood loss [30,31]. The administration was performed using an infusion pump Aladdin AL-1000 (World Precision Instruments, INC., Sarasota, FL, USA) through a venous catheter connected to a syringe placed in an infusion pump. The infusion volume was 50% of the volume of blood withdrawn. The infusion rate was 30 mL/kg/min.

### 2.6. VRT Issues Preparation

#### 2.6.1. Preservative/Anticoagulant Preparation

The preservative/anticoagulant was prepared according to the following formula: sodium citrate dihydrate—2.2 g, citric acid anhydrous extrapure—0.73 g, dextrose monohydrate extrapure AR, ACS, ExiPlus, Multi-Compendial—2.45 g, water for injection—up to 100 mL. Preparation was performed under aseptic conditions. After preparation, the solution was filtered through syringe-driven, 0.22 µm EPE204030, BIOFIL, China.

#### 2.6.2. Preparation of Autologous Blood for TF

Autologous blood for transfusion therapy was prepared as follows: during the HS model, blood was collected into a sterile syringe containing a preservative/anticoagulant equal to 10% of the volume of blood being collected. The syringe containing the blood and preservative was placed in an incubator at 37 °C after each blood collection and stored there for one hour after collection until the time of TF.

### 2.7. AP and HR Registration

AP and HR were recorded 30 min after surgical catheter implantation before blood sampling, immediately after blood sampling (HS start—15 min after the start of blood sampling), 60 min after blood sampling before VRT, 24 h after HS, and 48 h after HS (before catheter removal). AP was measured in 5-min intervals at each time point, with subsequent averaging. AP and HR were recorded through a carotid artery catheter connected to a PowerLab 4/35 computerized system with LabChart 7 Pro software (ADInstrument Inc., Bella Vista, New South Wales, Australia).

### 2.8. Blood Sample Collection

Blood was collected from animals through an arterial catheter before HS, 1 h after the end of HS before the start of VRT, 24 h after HS, and 48 h after HS before catheter removal. Blood samples were also collected from the inferior vena cava during necropsy on day 8 after HS and on day 29 after HS. In intact animals, blood was collected only during necropsy on days 8 and 29. The blood sampling scheme is shown in Table 2.

### 2.9. Removal of Catheters 48 h After HS

Forty-eight hours after HS, after a 5-min recording of AP and HR, blood sampling for hematology, hemostasis, and blood gas analysis was performed; animals were anesthetized with isoflurane, and venous and arterial catheters were removed. The vessels were ligated after catheters were removed, and skin at incision sites on the neck and withers was sutured. Following the surgical procedure, animals were placed in their holding cages.

### 2.10. Blood Gas and Metabolite Analysis

Blood samples collected from the animals’ arterial catheters before HS, before VRT, and 24 and 48 h after HS were analyzed using an ABL90 Flex gas analyzer (Radiometer Medical ApS, Copenhagen, Denmark). A 75-μL safeCLINITUBES capillary (Radiometer Medical ApS, Copenhagen, Denmark) was filled with whole blood collected from the animals’ arterial catheters and connected to the ABL90 Flex gas analyzer. The parameters listed in Table 3 were measured in the blood.

### 2.11. Body Temperature Registration

Animals’ body temperature was measured using an Omron Eco Temp Basic MS-246 electronic thermometer (Omron Healthcare Co., Ltd., Shanghai, China) before the start of HS, before VRT (60 min after the start of HS), after 1 h, after 3 h, after 24 h, and after 48 h after HS. To record body temperature, the thermometer tip was treated with petroleum jelly and inserted into the rectum.

### 2.12. Hematology Analysis

Whole blood samples (200 μL) were placed in Microvette^®^ 200 K3E tubes containing K3EDTA (SARSTEDT AG & Co., Nümbrecht, Germany). Blood was analyzed using a Mythic 18 hematology analyzer with a built-in veterinary program (C2 DIAGNOSTICS S.A., Montpellier, France) for the following parameters:
Red blood cell count (RBC) Hemoglobin level (Hb)Hematocrit (HCT)White blood cell count (WBC)Mean corpuscular hemoglobin content (MCH)Mean corpuscular hemoglobin concentration (MCHC) Mean corpuscular volume (MCV)Red cell distribution width—variation coefficient (RDW)Red cell distribution width-standard deviation (RDW-SD)Platelet count (PLT)Mean platelet volume—variation coefficient (MPV)Plateletcrit (PCT)Platelet distribution width—variation coefficient (PDW)Lymphocytes (LYM) Monocytes (MON) Granulocytes (GRA) 

The analysis was performed before HS, one hour after the start of HS before VRT, three hours after VRT, 24 h after HS, 48 h after HS, 8 days after HS, and 29 days after HS.

### 2.13. Hemostasis Analysis

Hemostasis analysis was performed on freshly collected plasma. To obtain plasma, 1 mL of blood was placed in a tube containing 100 μL of EDTA and centrifuged in an Eppendorf 5804 R centrifuge at 24 °C (3000 rpm, 15 min). Plasma was analyzed for activated partial thromboplastin time (APTT), prothrombin time (PT), and fibrinogen (Fg). Analysis was performed at the following time points: before HS, 24 h, 48 h, 8 days, and 29 days after HS.

### 2.14. Registration of Mortality and Assessment of Animal Welfare

Throughout the intravital phase of the study, animals were examined daily, assessing their general condition and recording signs of deviations in health and mortality.

### 2.15. Registration of Body Weight Gain

During the intravital phase, body weight of the animals was recorded weekly, and the increase in body weight was calculated as a % change in body weight on the day of measurement from the initial weight measured on the first day of the study (before HS).

### 2.16. Euthanasia

Animals were euthanized by anesthesia with a Vesotil^®^/Xyla^®^ mixture (30 mg/kg + 10 mg/kg, intramuscularly) followed by terminal blood sampling from the caudal vena cava. Half of the animals were euthanized on day 8 of the study, and half on day 29 of the study (Table 1).

### 2.17. Necropsy and Organ Weighing

During euthanasia, the animals’ body cavities and internal organs were examined, weighed, and then preserved in formalin for histological analysis. The following organs were collected: spleen, adrenal glands, kidneys, submandibular lymph nodes, thymus, heart, lungs, and brain. Relative organ weight was calculated as a percentage of body weight at necropsy.

### 2.18. Bone Marrow Smear and Myelogram Analysis

During necropsy on days 8 and 29 of the study, bone marrow was isolated from all surviving animals by washing it from the femur with a mixture of PBS (pH = 7.4) and fetal bovine serum (1:1) followed by centrifugation for 1 min in a Mini Spin centrifuge (Eppendorf, Hamburg, Germany) at 5000 rpm. Bone marrow smears were prepared from the resulting sediment and stained using the May-Grünwald/Romanovsky/Pappenheim method [52]. Stained smears were analyzed for hematopoietic cells—lymphoid, granulocyte/myeloid, and erythroid—by calculating the M:E (myeloid-to-erythroid ratio). Cells were counted using a 500-cell SFC “MINILAB” formed element counter. Slides were analyzed using a Leica DM LA light microscope (Leica, Wetzlar, Germany) at 40x magnification (in oil immersion).

### 2.19. Histology

Biomaterial samples were fixed in 10% neutral formalin, rinsed in running tap water, dehydrated in ascending alcohol concentrations, and embedded in paraffin. Paraffin sections (4–5 µm thick), stained with hematoxylin and eosin, were examined using light microscopy on an AxioScope A1 microscope (Carl Zeiss, Oberkochen, Germany). Micrographs of histological preparations were obtained using an Axiocam 305 color high-resolution camera (Carl Zeiss, Oberkochen, Germany) and ZEN 2.6 lite software (Carl Zeiss, Oberkochen, Germany). Table 4 shows the scoring scale used to assess the severity of pathohistological changes.

### 2.20. Statistics

Data are presented as mean values ± standard deviation (SD) or standard error of the mean (SEM), calculated in Excel. Statistical data processing was performed using Statistica for Windows v.7.0 software. Repeated measures ANOVA followed by a post-hoc Fisher LSD test was used to assess intergroup differences in values measured dynamically (hemodynamics, body temperature, hematology, hemostasis, oximetry, body weight gain). One-way ANOVA followed by a post-hoc Fisher LSD test was used to analyze intergroup differences for values measured at necropsy (organ weight, semiquantitative assessment of histological pathology, myelogram analysis results).

## 3. Results

### 3.1. Mortality

Of 10 animals that received SA as VRT, 2 animals died within the first three hours after HS. On day 8, half of the surviving animals in this group, i.e., 4 out of 8, were euthanized. One more animal (1 out of 4) in this group died on day 20 after HS. No deaths were observed among animals that received AB after HS. This fact leads to an important remark that must be taken into account when evaluating all the parameters studied in this study: not all samples were obtained from deceased animals and, therefore, could not be evaluated; only parameters from surviving animals whose condition did not deteriorate to the terminal stage were evaluated.

### 3.2. Weight Gain

**Figure 2:**
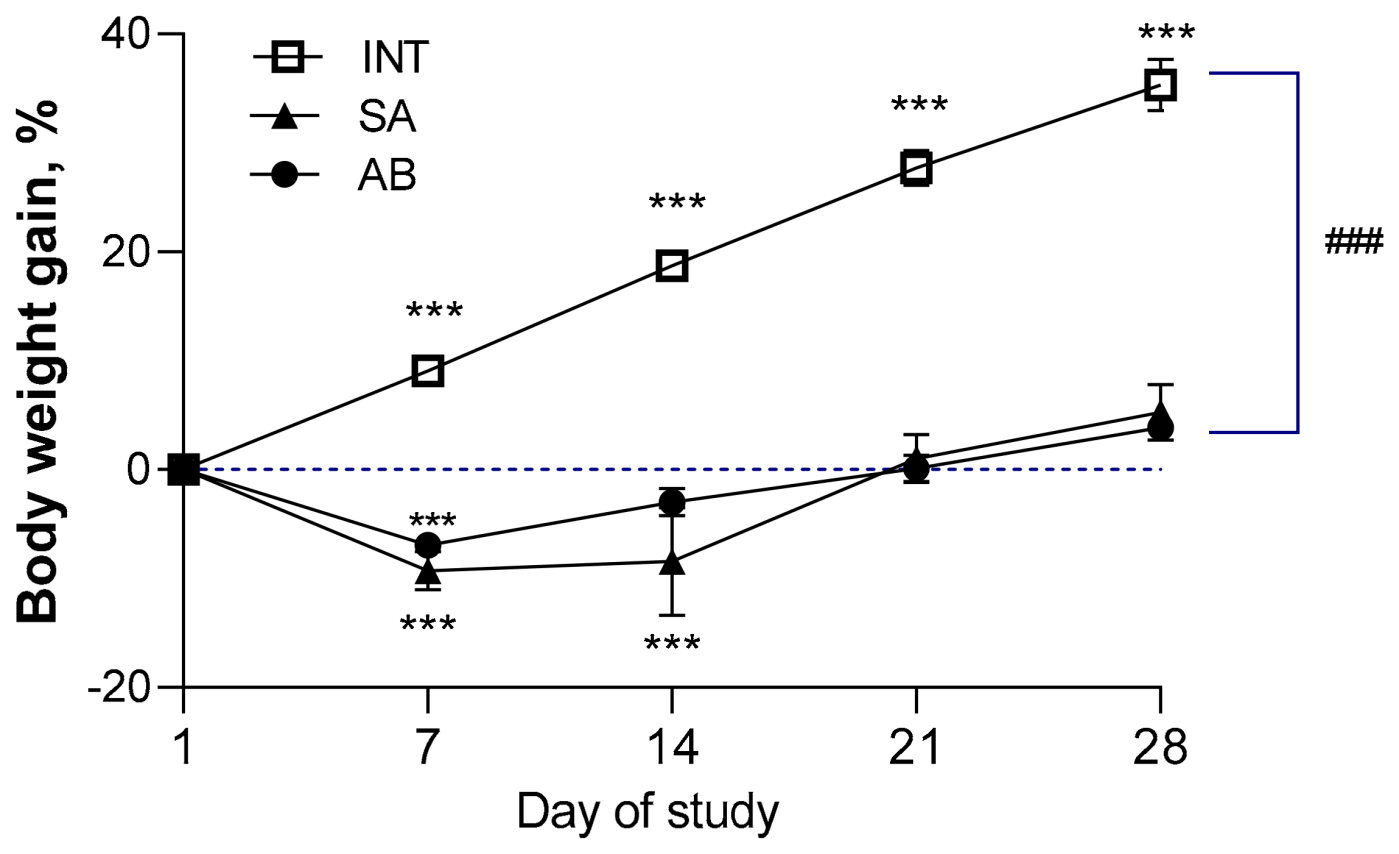
Body weight gain calculated as % of body weight at necropsy. *** *p* ≤ 0.001 relative to baseline values on day 1, ### *p* ≤ 0.001 relative to INT group (in the figure, statistically significant differences are indicated by brackets) according to Repeated measures ANOVA, post hoc Fisher LSD test.

All animals lost weight after HS, and their body weight gain was significantly reduced compared to intact animals throughout the observation period. This did not differ between the groups receiving SA and AB after HS. Data are presented in Figure 2 and Table 5.

### 3.3. AP and HR

**Figure 3:**
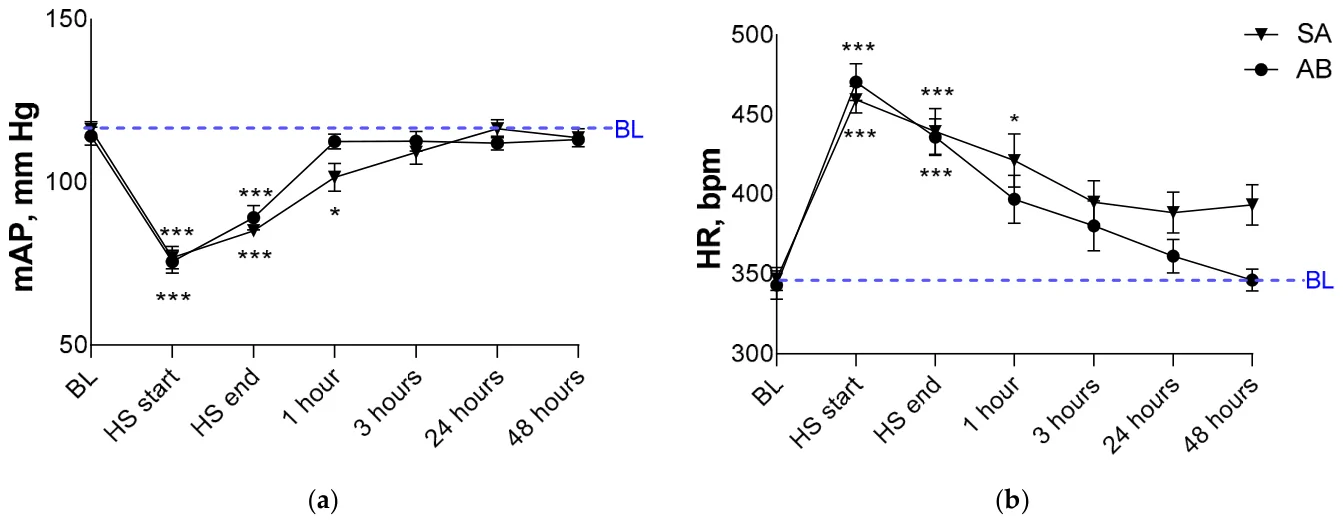
Hemodynamic parameters in the first 48 h of the experiment. (**a**) mean arterial pressure (mAP) and (**b**) heart rate (HR). Measurement points: BL—baseline level before the start of HS, HS start—10 min after the start of HS, HS end—60 min after the start of HS, 1 h—1 h after VRT, 3 h—3 h after VRT, 24 h—24 h after HS, 48 h—48 h after HS. * *p* ≤ 0.05, *** *p* ≤ 0.001 relative to BL according to Repeated Measures ANOVA, post-hoc Fisher LSD test.

Ten minutes after the start of blood sampling, blood pressure dropped by more than 30% relative to baseline values, while heart rate increased by 30%. The drop in blood pressure, coupled with a compensatory increase in heart rate, continued for three hours in the SA group and for one hour in the AB group. Thus, AB was more effective in restoring hemodynamic parameters after HS (Figure 3).

### 3.4. Body Temperature

**Figure 4:**
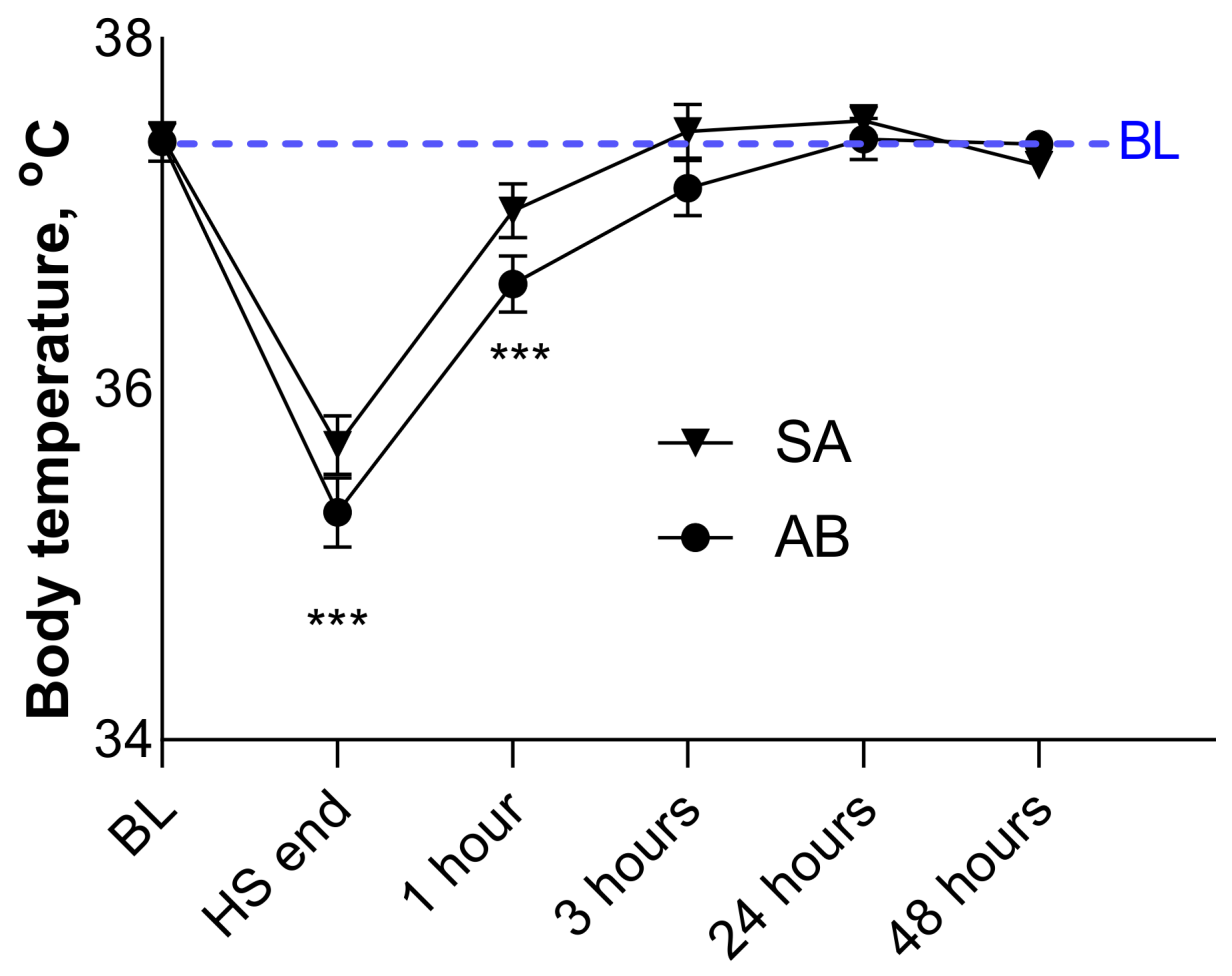
Dynamics of animal body temperature during HS modeling. Measurement points: BL—baseline before HS start, HS end—60 min after HS start, 1 h—1 h after VRT, 3 h—3 h after VRT, 24 h—24 h after HS, 48 h—48 h after HS. *** *p* ≤ 0.001 relative to BL according to Repeated measures ANOVA, post hoc Fisher LSD test.

Body temperature significantly decreased from 37.5 °C to 35.5 °C in animals 60 min after the start of HS, followed by recovery to baseline values 3 h after HS (Figure 4).

### 3.5. Registration of Blood Gases and Metabolites with a Gas Analyzer (Oximetry)

**Figure 5:**
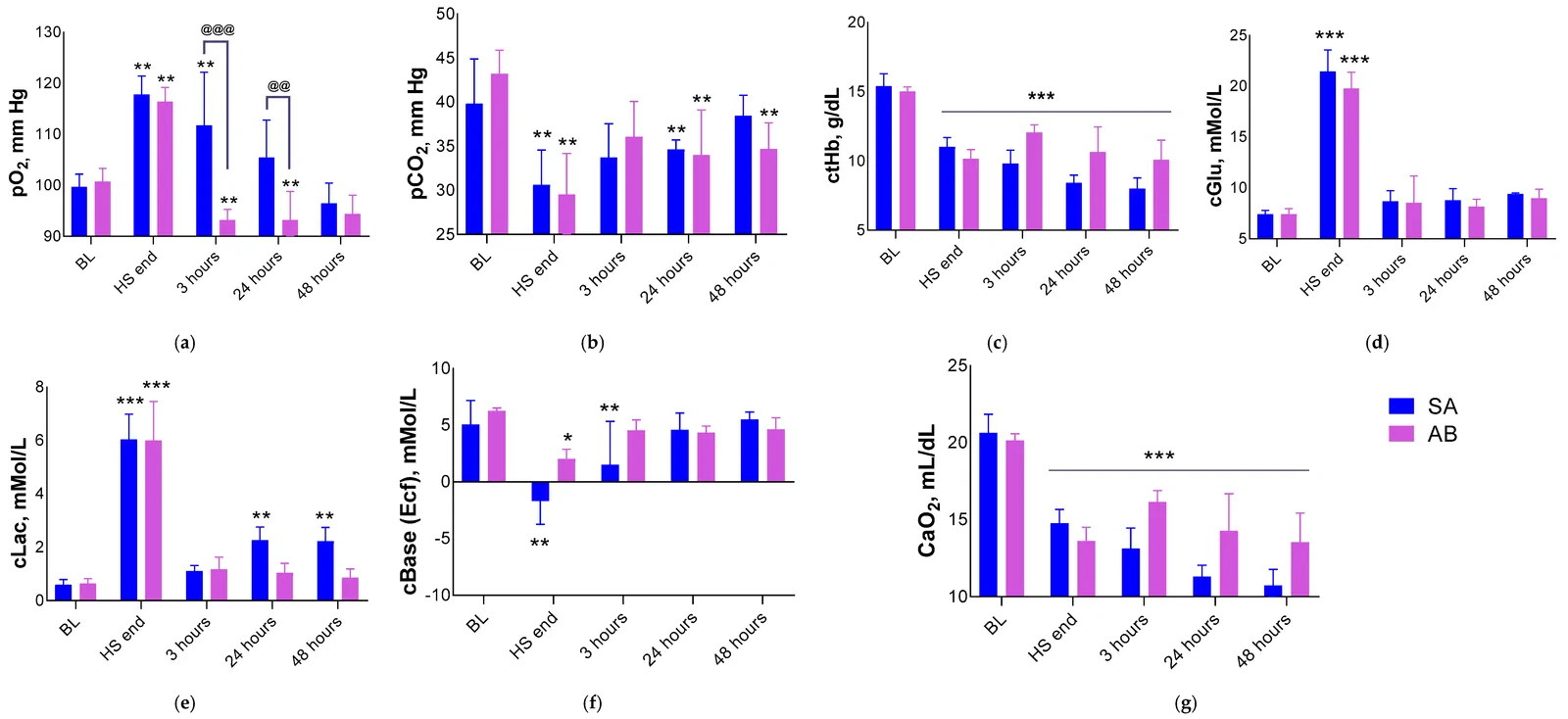
Whole blood oximetry parameters during the first 48 h of the experiment. (**a**) partial pressure of oxygen (pO_2_), (**b**) partial pressure of carbon dioxide (pCO_2_), (**c**) total hemoglobin concentration (ctHb), (**d**) glucose concentration (cGlu), (**e**) lactate anion concentration (cLac), (**f**) Standard Base Excess of Extracellular Fluid (cBase (Ecf)), (**g**) arterial oxygen content (CaO_2_). Data are presented as MEAN + SD. * *p* ≤ 0.05, ** *p* ≤ 0.01, *** *p* ≤ 0.001 relative to BL, @@ *p* ≤ 0.01, @@@ *p* ≤ 0.001 relative to the SA group according to Repeated measures ANOVA with post-hoc Fisher LSD test.

The oximetry parameters that changed during the experiment are shown in Figure 5. Sixty minutes after the initiation of HS, the animals’ pO_2_ increased, indicating an increase in the amount of oxygen dissolved in blood plasma. After 3 h, pO_2_ in animals receiving AB decreased below baseline and remained reduced for up to 24 h. In the animals receiving SA, pO_2_ remained elevated for up to 3 h after VRT, with pO_2_ values being significantly higher than in animals receiving AB at 3 h and 24 h after HS. pCO_2_ decreased after HS and remained reduced for up to 48 h after HS. ctHb also decreased after HS and remained reduced for up to 48 h, with no differences observed between SA and AB. cGlu increased sharply fivefold after 60 min of HS, returning to baseline values within 3 h after HS in both model groups. cLac increased eightfold after 60 min of HS, returning to normal by 3 h after HS in the AB-treated group, while in the SA-treated group it decreased but did not return to baseline, remaining twofold elevated for up to 48 h after HS. cBase (Ecf), reflecting the metabolic component of blood buffering capacity, decreased in animals 60 min after HS, which correlates with an increase in blood cLac at the same time. The calculated CaO_2_ indicator decreased in animals after HS and remained reduced for up to 48 h after HS.

### 3.6. Hematology

The dynamics of hematological parameter changes are presented in Table 6. After HS, all animals experienced a decrease in RBC count, cHb, and HCT. This decrease persisted throughout the 28 days of the study, although it was less pronounced in animals receiving AB. On days 8 and 29 after HS, animals showed an increase in the red RDW, indicating increased variability in red blood cell size in the bloodstream. Judging by the increase in MCV at these same times, more young, large RBCs, which had matured in the bone marrow, appeared in the blood. Furthermore, animals after HS experienced a gradual increase in WBC count—in the first 24 h due to an increase in GRA, and from 48 h to day 8 of the study, due to ongoing lymphocytosis.

On the 8th day after HS, a twofold increase in PLT count was observed, caused by the release of new cells from the bone marrow after maturation, indirectly confirmed by a decrease in the MPV (typical of young cells). Moreover, both before and after the 8th day, PLT counts did not differ significantly from baseline, indicating that excess PLT were deposited in the spleen. These changes were less pronounced in the group of animals receiving AB.

### 3.7. Myelogram

**Figure 6:**
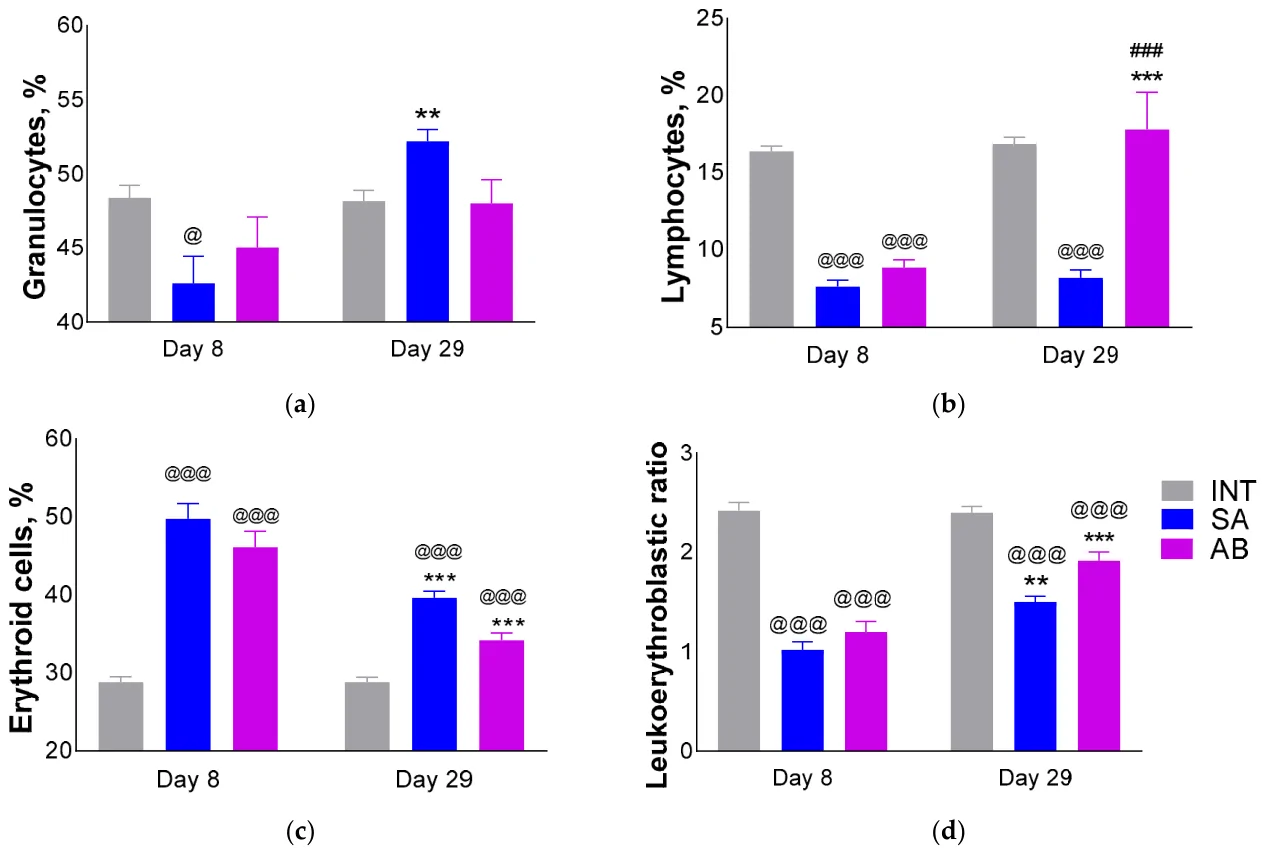
Myelogram on days 8 and 29 after HS. (**a**) Granulocytes,%; (**b**) Lymphocytes, %; (**c**) Erythroud cells, %; (**d**) Leucoerythroblastic ratio. ** *p* ≤ 0.01, *** *p* ≤ 0.001 relative to BL, @ *p* ≤ 0.05, @@@ *p* ≤ 0.001 relative to the intact group, ### *p* ≤ 0.001 relative to the SA group according to Repeated measures ANOVA with post-hoc Fisher LSD test. Data are presented as arithmetic mean + standard error of the mean.

Figure 6 shows the cellular composition of animal bone marrow. The granulocyte count was significantly reduced in animals infused with SA on day 8 post-HS, with recovery by day 29 post-HS. The granulocyte count in animals treated with SA was higher than BL on day 29 post-HS, which may indicate a persistent inflammatory process. A significant, almost threefold, drop in the lymphocyte count was observed on day 8 post-HS, which persisted until day 29 in animals treated with SA but fully recovered in animals treated with AB. A twofold increase in the erythroid count was observed on day 8 post-HS, with a recovery by day 29 post-HS. The calculated leukoerythroblastic ratio was reduced on day 8 post-HS, with a recovery by day 29, although the recovery was not complete.

### 3.8. Hemostasis

**Figure 7:**
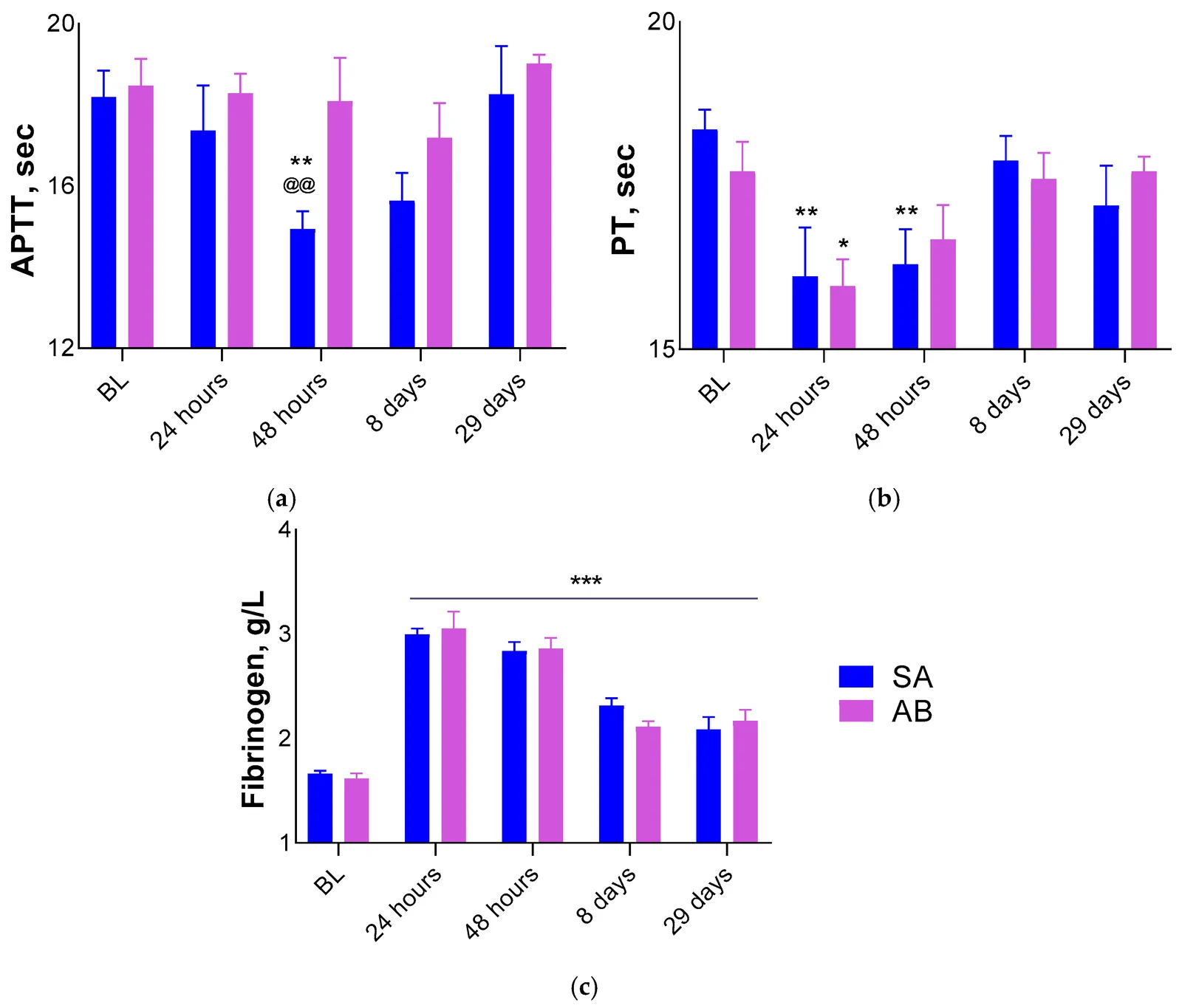
Changes in hemostasis parameters. (**a**)—partial activated thromboplastin time (APTT), (**b**)—prothrombin time (PT), (**c**)—fibrinogen concentration. * *p* ≤ 0.05, ** *p* ≤ 0.01, *** *p* ≤ 0.001 relative to BL, @@ *p* ≤ 0.01 relative to the SA group according to Repeated measures ANOVA with post-hoc Fisher LSD test. Data are presented as mean + standard error of the mean.

In animals treated with SA (but not AB) after HS, APTT decreased 48 h after HS. PT decreased relative to BL 24 h after HS in the SA and AB groups, remaining decreased after 48 h only in the SA group. On days 8 and 29 after HS, both parameters recovered to baseline levels. Fibrinogen concentration was elevated in both groups at all time points (24 h, 48 h, 8 days, and 29 days after HS). Data are shown in Figure 7.

### 3.9. Organ Weight

After HS, animals’ organ weights changed relative to those in intact animals. Spleen weight increased on day 8 after HS (more markedly in the SA-treated group), followed by a recovery-like decrease by day 29. Kidney weight decreased on day 8 and remained reduced on day 29 after HS. Liver weight increased on day 8 and then decreased on day 29 after HS. Thymus weight decreased significantly—almost fivefold—on day 8 in animals after HS. Although some recovery-like increase in thymus weight was observed by day 29, the organ weight remained almost twofold lower than in intact animals. Organ weight data are presented in Table 7.

### 3.10. Histology

**Figure 8:**
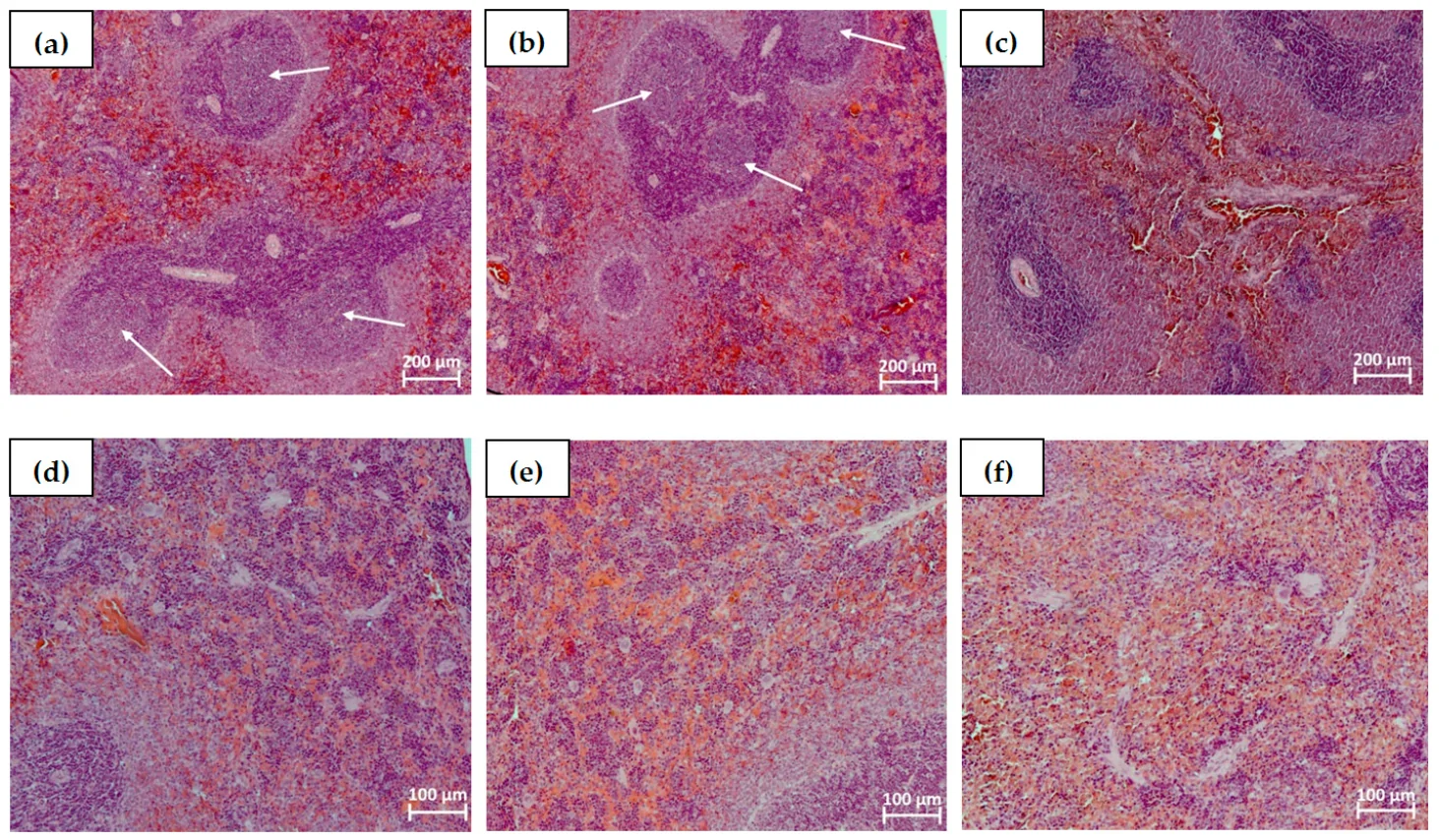
Spleen fragments from male SD rats on day 8. Hyperplasia of white pulp cells and numerous large germinal centers in lymphoid follicles (white arrows) in animals received SA (**a**) and AB (**b**), white pulp of an intact animal (**c**). Enhanced extramedullary hematopoiesis in red pulp in the SA group (**d**) and in the AB group (**e**). Normal background extramedullary hematopoiesis in the red pulp of the spleen of an intact animal (**f**). Stained with hematoxylin and eosin (**a**–**c**). Magnification 50× (**d**–**f**). magnification 100×.

On the 8th day after HS, moderate hyperplasia of white pulp cells with presence of large germinal centers in lymphoid follicles was observed in the spleen of all animals receiving SA (Figure 8a), while an increase in extramedullary hematopoiesis was noted in the red pulp of the organ (Figure 8d). Following the administration of AB, identical histopathological changes of similar severity were observed in the spleen (Figure 8b,e). By the 29th day of the study, animals from groups receiving SA and AB showed a decrease to a moderate degree of hyperplasia of white pulp cells of the spleen, with a decrease in the size of germinal centers in lymphoid follicles, while extramedullary hematopoiesis in the red pulp decreased to normal background values.

**Figure 9:**
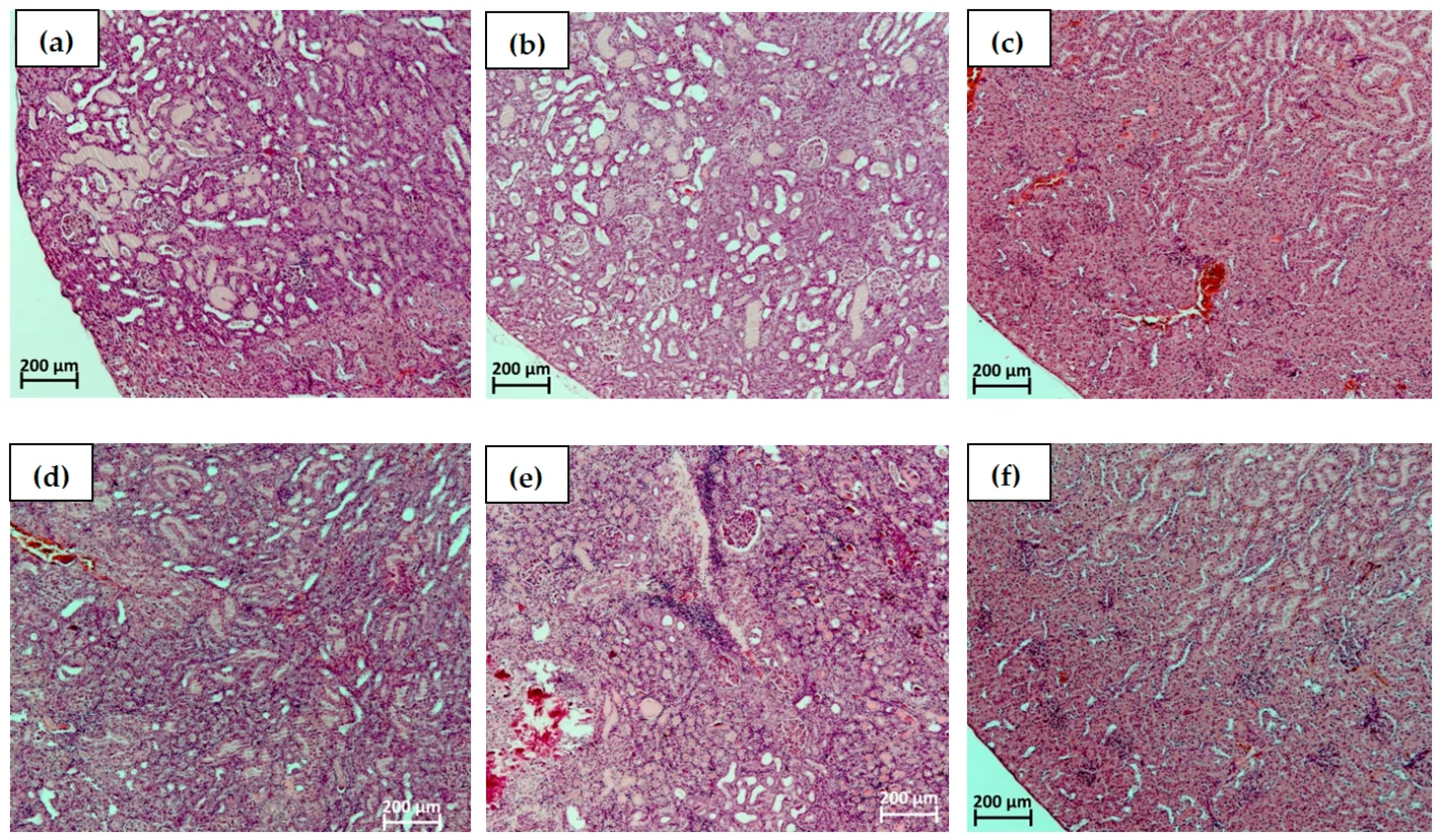
Kidney fragments of male SD rats. On the 8th day, signs of acute focal wedge-shaped ischemia of the medulla with preservation of the glomerular apparatus and necrotic changes in epithelial cells of renal tubules were observed in animals with SA (**a**) and AB (**b**), a fragment of a kidney of an intact animal on the 8th day (**c**). On the 29th day, focal post-ischemic nephrosclerosis was observed in the SA group (**d**) and in the AB group (**e**). Kidney fragment of an intact animal on the 29th day—(**f**). Stained with hematoxylin and eosin, magnification 50×.

In the kidneys of half of the male rats on the 8th day after HS with SA, signs of acute organ ischemia were observed: a wedge-shaped ischemic focus with the preservation of the glomerular apparatus and necrotic changes in epithelial cells of renal tubules (Figure 9a). Identical changes were observed in the majority of animals with AB TF (Figure 9b). On the 29th day of the study, focal post-ischemic nephrosclerosis was observed in the kidneys of one of three surviving animals receiving SA (Figure 9d), while in the group of animals receiving AB, in which all five animals survived to the 29th day, a similar phenomenon was observed in two animals (Figure 9e).

**Figure 10:**
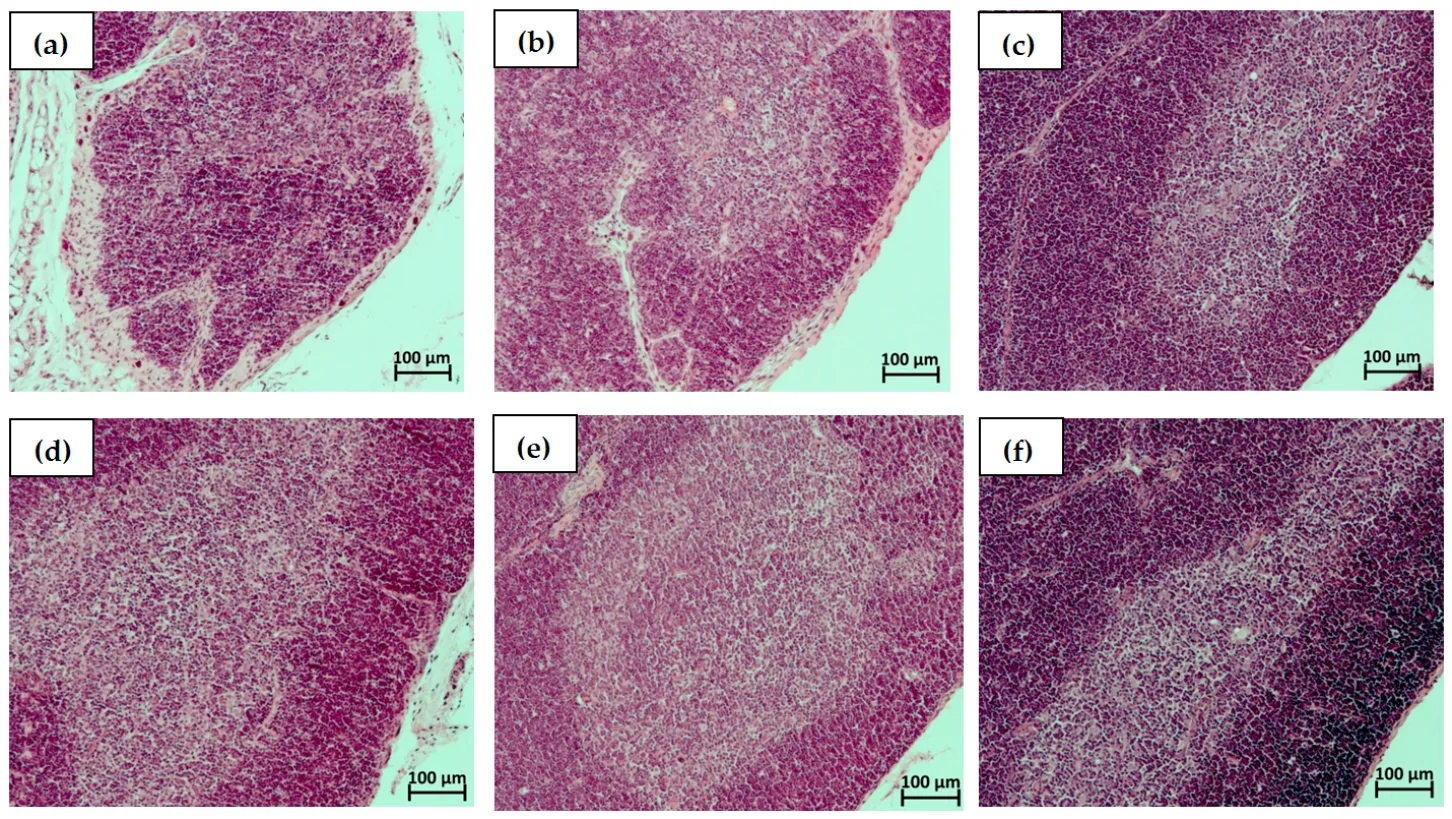
Thymus fragments from male SD rats. On the 8th day after HS, pronounced accidental involution with erasure of the features of the histological structure of the organ was observed in the SA group (**a**), in the AB group, a moderate decrease in corticomedullary ratio was observed due to a reduction in the number of lymphoid cells in the cortex of lobules, with a weak manifestation of accidental involution (**b**); thymus of intact animal (**c**). On the 29th day after HS, a slightly reduced corticomedullary ratio was observed in lobules of the organ in the SA group (**d**), in the AB group (**e**); thymus of the intact animal (**f**). Stained with hematoxylin and eosin. Magnification 100×.

Pronounced changes were observed in the thymus of animals after HS. On the 8th day, all animals administered SA showed signs of accidental involution and a marked decrease in corticomedullary ratio due to a reduction in the number of lymphoid cells of the lobular cortex. Similar changes were observed after AB administration, but they were significantly less pronounced based on a semiquantitative assessment (Figure 10a,b, Table 8). On the 29th day after HS, a slightly reduced corticomedullary ratio in the thymus of all animals in both model groups was observed (Figure 10d,e, Table 9).

**Figure 11:**
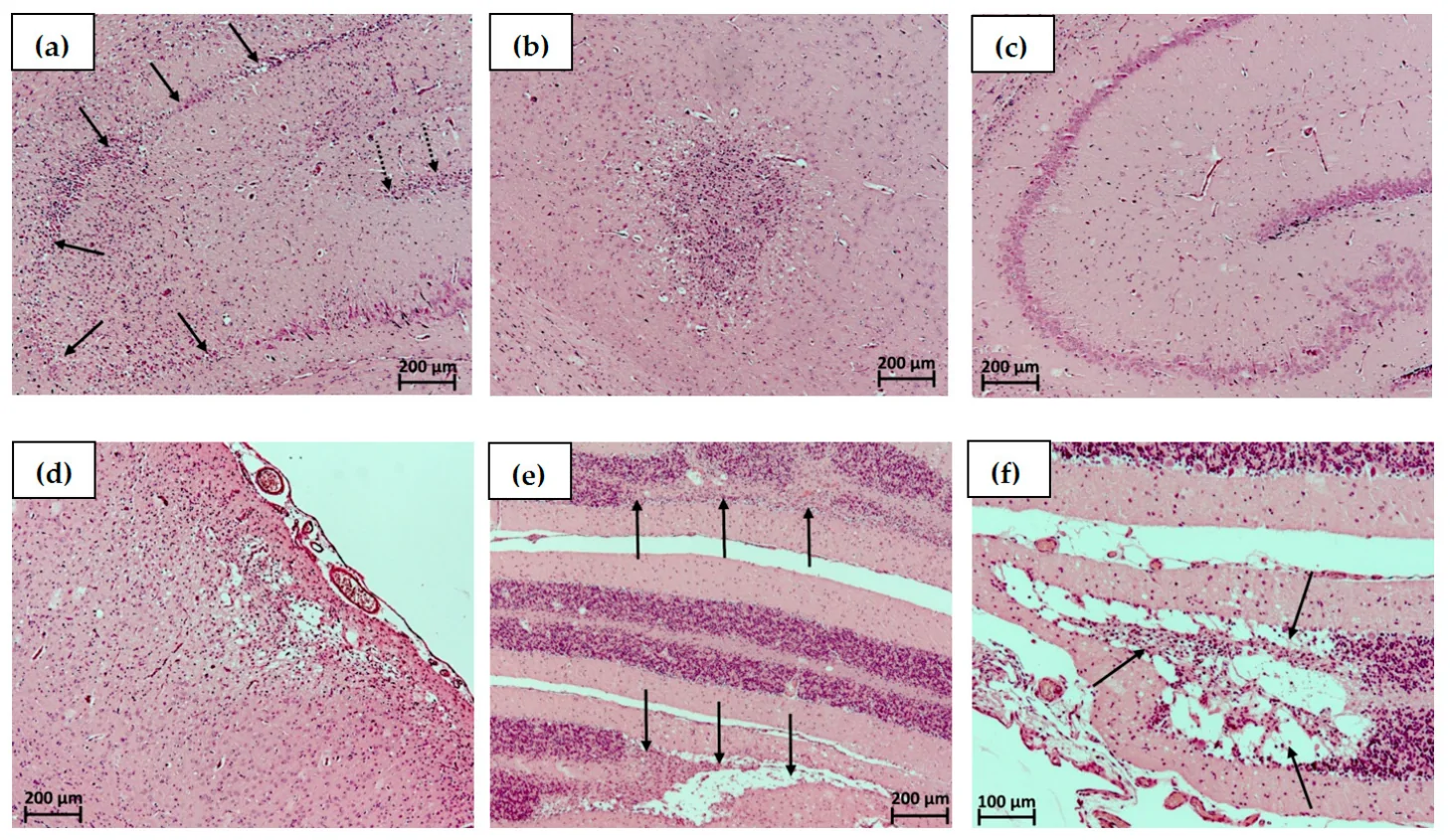
Brain fragments of male SD rats. On day 8 after HS, one animal from the SA group showed massive neuronal loss with a glial reaction in the CA2-CA3 projection (solid arrows) and dentate gyrus (dashed arrows) of the hippocampus (**a**). Also on day 8, one animal from the AB group showed a small focus of necrosis with a glial reaction in the projection of the basal ganglia (in the center of the micrograph)—(**b**). Normal histological structure of the hippocampus of an intact animal (**c**). On day 29 after HS, pathological changes were observed in one animal from the SA group; the next three micropreparations are from this animal. (**d**)—small gliomesodermal scar in the projection of the parietal cortex, (**e**,**f**)—small gliomesodermal scars in cerebellar cortex at sites of previous ischemic necrosis (arrows). Stained with hematoxylin and eosin. Magnification 50× (**a**–**e**) and 100× (**f**).

On the 8th day after HS modeling, massive neuronal loss with a glial reaction was observed in the CA1, CA2, CA3, and dentate gyrus projections of the hippocampus in individual animals treated with SA and AB (Figure 11a). In one animal treated with SA, a small focus of necrosis with a glial reaction was found in the projection of the basal ganglia (Figure 11b). On the 29th day, in one animal treated with SA (out of three surviving animals), formed gliomesodermal scars were found in the parietal cortex (Figure 11d) and cerebellar cortex (Figure 11e,f) at sites of previous ischemic necrosis of nervous tissue.

A description of histological changes with their semiquantitative scoring is given in Table 8 and Table 9.

## 4. Discussion

The severity of blood loss in humans is classified based on the volume of blood lost [1] as follows: hemorrhage class I (blood loss <15% of the CBV), class II (blood loss 15–30%), class III (blood loss 30–40%), class IV (blood loss ≥40%). HS develops with blood loss of classes III–IV and is characterized by critical inclusion of compensatory mechanisms, including sympathetic activation, tachycardia, inflammatory cascades, and tissue hypoperfusion, leading to an increase in lactate anion, base deficiency, persistent shock, accompanied by inflammatory activation and rapid increase in multiple organ failure [53,54,55]. In preclinical practice, HS/blood loss is most often modeled on anesthetized animals by collecting blood until blood pressure drops to a certain level (30–40 mm Hg) [38,39,56,57], which most closely correlates with intraoperative blood loss. In our study, we used a different approach, collecting a strictly defined volume of blood (40% of CBV) from awake animals. This method is, firstly, more relevant to conditions associated with traumatic blood loss and, secondly, more standardized, as a standard volume of blood is collected from the animals. In our study, the drop in pressure occurred at an early stage of the onset of blood loss and was compensated within 1 h after the onset of blood loss, accompanied by a compensatory increase in heart rate, which indicates an increase in venous return [58], baroreflex promotion, and vasoconstriction caused by sympathetic activation [59]. Modeling of HS in anesthetized rats using the fixed-pressure hemorrhage method is characterized by a drop in AP without a compensatory increase in HR [60], and even with a drop in HR [57], which is due to the suppression of the baroreflex and the sympathetic component, which plays one of the key roles in the development of the pathological process in HS [54,55]. In addition, with fixed-pressure HS, the researcher focuses on the blood pressure value, which is quite labile due to the uneven activation of compensatory mechanisms, such as the release of interstitial fluid into the bloodstream, venous return, and the ejection of blood from blood depots. To maintain a stably low AP value for 1–2 h, after reaching the target AP value (usually 40–60 mm Hg), it is necessary to constantly either withdraw or, conversely, administer different volumes of blood [38,39,60], which does not allow for the unification of the impact of blood loss on the body, leading to high variability in the consequences of HS. Thus, our method of modeling HS by collecting a fixed volume of 40% of the CBV in awake animals activates most of the compensatory mechanisms, allowing a more accurate study of the body’s reactions to HS and a better assessment of the effectiveness of therapy.

It should be noted separately that modeling of combined conditions, for example, HS plus injuries of various localizations, is only possible under anesthesia [43,44].

In our study, we showed that animals develop hypothermia in the first hour of HS, which fully correlates with the processes occurring in clinical practice during the development of the so-called death triad—hypothermia, coagulopathy and acidosis [61]. Hypothermia develops as a result of hypoperfusion and metabolic disturbance of heat generation [1]. Since enzymatic reactions occur at normothermia, a decrease in temperature leads to a disruption in the functioning of the enzymes of the coagulation system [62]. In our study, 24 h after HS, the APTT, an indicator characterizing the intrinsic pathway of coagulation activation, decreased in animals receiving SA, indicating a disruption of the intrinsic coagulation pathway. This was not observed in animals receiving AB, confirming the results of whole blood transfusion studies [31]. PT decreases in both AS-treated and AB-treated animals. PT characterizes the extrinsic pathway of coagulation activation, and its decrease indicates pathological hypercoagulation and an increased risk of thrombosis. In humans, trauma associated with massive blood loss results in coagulopathy, manifested by the same changes in coagulation parameters [63]. Post-traumatic coagulopathy is also indicated by elevated levels of Fg—coagulation factor I, which is also an acute-phase reactant. In our study, Fg levels remained elevated for 29 days after HS. The hyperfibrinemia we observed, in addition to post-traumatic coagulopathy, indicates compensatory neurohumoral processes, an enhanced immune response, and post-traumatic procoagulant readiness of the body [64,65].

In our study, we used a strategy of restrictive VRT and maintaining permissible hypotension. We selected the volume of VRT based on the results of retrospective human studies, which showed that the survival rate of patients with traumatic hemorrhage was inversely proportional to the volume of VRT. For example, a retrospective analysis of a German database of 17,200 patients with multiple trauma showed that dilutional coagulopathy developed in patients with increasing infusion volume (from 40% with an infusion volume of 2000 mL to 70% with an infusion volume of 4000 mL) [17]. Another study [23] demonstrated a dependence of survival in patients with polytrauma on volume of infusion: with an increase in the volume of infusion above 1500 mL, survival decreased. These data are confirmed by a prospective clinical study performed on patients with penetrating trauma and HS, in whom the incidence of coagulopathy increased with an increase of infusion volume [66]. Observed lymphocytosis, developing 24–48 h after HS, indicates a massive release of dormant mature lymphocytes from lymphoid depots—mesenchymal lymph nodes and the spleen—due to their activation under the influence of acute hypoxia and stress. However, the inevitable apoptosis of these immunocompetent cells causes the development of systemic inflammatory response syndrome (SIRS) and sepsis [67,68,69,70]. Granulocytosis, which develops within 3 h and persists for up to 24 h after HS, is caused by two factors: the release of GRA from blood reservoirs (liver and spleen) and the release of leukocytes from the bone marrow due to stimulation of the systemic inflammatory response both in response to shock and in response to VRT, leading to reperfusion stress with the release of reactive oxygen species and activation of the immune response [71,72,73]. 48 h after HS, inflammation persists, but is less pronounced after AB transfusion. A study by Spinella et al. showed that warm fresh whole blood transfusion is associated with improved survival for patients with combat-related traumatic injuries [31,74]. In our model, the number of RBCs fell proportionally to the volume of withdrawn blood, that is, by 40%, while in the AB group, after TF, it increased in proportion to the returned volume of whole blood. The same changes occurred with Hb content and HCT. The decline in RBC count, Hb content, and HCT persisted until day 29 after GS, indicating the development of anemia, a characteristic consequence of massive blood loss [75], The observed increase in erythrocyte anisocytosis, namely an increase in the number of large cells, accompanied by an increase in the hemoglobin content in the blood and in erythrocytes on the 8th day after HS, indicates the release of young erythrocytes from the bone marrow after the completion of the hematopoiesis cycle [76]. Although the erythroid lineage in our study was stimulated with a nearly twofold increase in the relative number of erythroid cells in the bone marrow on the 8th day after HS, the decrease of RBC and Hb content in blood persisted until the end of the study (29th day after HS). This demonstrates that anemia that developed in animals after HS persists, similar to what occurs in humans after HS, despite therapy [77,78,79].

More than twofold increase in PLT count observed in our study, with a predominance of small, young cells, on day 8 after HS, indicates the release of new PLT after the detachment of new cells from HS-activated megakaryocytes in the bone marrow and their release into the bloodstream. This phenomenon is explained by the development of SIRS under the influence of HS-activated inflammatory stimuli [80]. Thus, in patients with trauma, an increase in platelet-activating factor (PAF) and IL-8, responsible for activation of thrombopoiesis and granulopoiesis, was shown [81]. Also, budding of new PLT from megakaryocytes is activated by heme-containing molecules [82]. These PLT in turn activate monocytes (macrophage precursors) [83], which was also shown in our study on the 8th day after HS.

In humans, with HS, there is a mobilization of hematopoietic progenitor cells (HPCs) in humans, and in severe HS, the mobilization of HPCs into blood from bone marrow is associated with bone marrow dysfunction [73,84].

Our study confirms the findings of studies conducted on patients with HS. Specifically, we observed a decrease in GRA and LYM levels in the bone marrow on day 8 in animals that underwent VRT with SA. Moreover, administration of AB prevented a decrease in GRA levels, but not LYM, in the bone marrow. On day 29, the GRA count increased in animals receiving SA, and the LYM count in the bone marrow remained significantly low, while in animals receiving AB, the number of bone marrow LYM increased. Furthermore, hyperstimulation of the erythroid lineage in the bone marrow persisted until day 29 after HS in both groups, reducing the leukoerythroblastic ratio. Myelogram examination demonstrates the development of bone marrow dysfunction after HS and the protective effect of AB on the GRA count in the bone marrow. In a study performed on patients with polytrauma and HS, on the 3rd day after HS, inhibition of hematopoietic stem cell growth in the bone marrow was shown [73,85,86,87], supporting relevance of our HS modeling findings to the condition developing in humans.

Increase in pO_2_ in arterial blood during the first hour of HS is due to increased respiratory rate, as well as decreased RBC and Hb levels. It is known that in venous blood, pO_2_ decreases during blood loss due to hypoxic redistribution of blood to organs and tissues most sensitive to hypoxia (brain, heart), and increased oxygen utilization by these organs [6,88]. In our study, we demonstrated that administration of AB completely compensates for the increase in arterial pO_2_ as early as 3 h after HS, unlike SA. We demonstrated that pCO_2_ decreases during HS, and even the administration of AB does not compensate for this decrease during the first two days after HS. Arterial pCO_2_ corresponds to that in pulmonary alveoli and depends on oxygen transport, which decreases during development of HS due to decreased cardiac output and tissue hypoperfusion, which causes a drop in pCO_2_. Furthermore, compensatory hyperventilation during HS contributes to the decrease in pCO_2_. Similar data were obtained in studies on pigs [89,90].

In our study, an explosive fivefold increase in cGlu during HS serves as an indicator of stress and indicates the body’s response known as “stress hyperglycemia.” Glucose released into the blood due to stress-induced activation of sympathetic glycolysis is intended to provide energy to vital organs (the brain and heart) and also draw interstitial fluid into the bloodstream to compensate for the drop in AP [90,91,92]. Acute lactatemia, which develops immediately after blood loss, indicates tissue hypoperfusion and a switch to an anaerobic metabolic pathway. Lactate levels correlate with the severity of shock and subsequent mortality [93,94]. An increase in cLac is associated with a decrease in the excess of bases (cBase (Ecf)), and in the case of the group of animals receiving SA, a more pronounced decrease in this indicator may be associated with the introduction of albumin, which is a weak acid [95,96,97].

Over the course of 48 h of blood gas recording after HS, we observed a significant drop in CaO_2_, consistent with a drop in ctHb and RBC count. This complex of changes indicates the development of anemia as a direct consequence of blood loss [98,99,100,101,102].

Due to hypoperfusion, anemia and metabolic disorders developing in HS, changes occur in organs and tissues most sensitive to hypoxia, such as the brain and kidneys, and organs involved in hematopoiesis, such as the spleen [103]. In our study, kidney weight decreased significantly on day 8 after HS and remained unchanged until day 29 after HS. Pathological focal ischemic changes were observed in the kidneys, progressing to necrosis in half of the cases. This correlates with pathological changes seen in humans with hypovolemic shock. The kidneys are extremely susceptible to blood loss and hypoxia, as they require a large amount of energy to maintain acid-base balance [104]. In addition, HS disrupts the filtration function of the kidneys, which directly depends on perfusion pressure [105]. HS leads to hypoperfusion and hypoxia in renal tubules and glomeruli, which leads to a pathological chain of ischemia-inflammation-acute renal failure [106,107]. At the same time, the juxtaglomerular apparatus of the kidneys, under the influence of hypoxia and, as a consequence, activation of the renin-angiotensin-aldosterone system, increases production of erythropoietin, which in turn stimulates erythropoiesis in bone marrow, as well as extramedullary hematopoiesis [108,109,110], which is confirmed by results of our study.

The spleen is a secondary lymphoid organ that plays a key role in blood filtration, as well as in innate and adaptive immune responses, actively responding to blood loss and hypoxia. In our study, we observed white pulp cell hyperplasia and numerous large germinal centers in lymphoid follicles of animals on day 8 after HS, associated with an increase in organ weight and development of an inflammatory response, also confirmed by pronounced leukocytosis. In a study on rats with HS, Warren et al., 2017, a decrease in the rate of oxygen consumption and ATP production in the mitochondria of splenocytes, and the inflammatory response was demonstrated by significantly increased expression of IL-6, IFN-β, Mip-1α, IL-10, and NFκbp65, indicating an inflammatory response of splenocytes to HS [41]. On the 29th day after HS, spleen weight in animals decreased to the level of control animals, while increased extramedullary hematopoiesis was observed in the red pulp; that is, formation of erythrocytes outside the bone marrow, which is a compensatory response of the body to blood loss and subsequent persistent anemia [110,111,112,113].

The thymus is an important lymphatic organ regulating immune function; it is involved in proliferation of hematopoietic stem cells, differentiation into T-lymphocytes, and maintenance of immune function; it is an organ sensitive to hypoxia and stress [114,115,116,117,118].

In our study, thymus weight on day 8 decreased fivefold in the SA group and threefold in the AB group. This weight loss was accompanied by pronounced accidental involution, that is, severe atrophy and massive destruction of immune cells in the cortex. This process is the thymus’s response to HS, a severe stress response that results in accidental organ involution, the release of T-lymphocytes into the blood, and active thymocyte apoptosis [119,120,121]. By day 29 after HS, the organ continues to lose weight, while involutional changes persist. The initial stage of thymus involution (up to day 8 after HS) is accompanied by an increase in the number of LYM in the blood, a marker of inflammatory process development.

The brain, as the organ most sensitive to hypoxia, suffers from HS of various origins [122,123]. In our study, pathological changes affected the hippocampus—the brain structure most sensitive to global ischemia. Massive blood loss due to hypoperfusion leads to depletion of energy resources, mitochondrial dysfunction, and neuronal death [124,125]. Ischemic changes in the hippocampus of animals were isolated and were corrected by the administration of AB.

All the described changes in organs should be considered taking into account the death of 30% of animals in the group receiving SA. It should also be noted that this study was designed to characterize the HS model, and VRT was not the subject of study, but was used as part of the model to confirm its validity. The VRT strategies used demonstrated the aptitude of our model, confirming previously obtained results, including those in humans.

To ensure a more complete presentation and interpretation of the study results, several limitations of our work should be noted. For example, our study used only male animals, which is common in experimental pharmacology, although using animals of both sexes would have improved the generalizability of the model. Also, the sample size was not large, especially considering the mortality in the SA group, thus limiting the number of samples available for analysis. Furthermore, this study did not include a group of sham-operated animals; however, this limitation was somewhat offset by the inclusion of an intact animal group, which served as a standard for assessing the dynamics of changes in animals with HS.

Thus, in our study, we demonstrated that blood withdrawal at a volume of 40% of CBV and an hourly deficit followed by partial VRT represent a successful model of HS. Development of HS is confirmed by results of analysis of hemodynamic, hemostasis, and body temperature, blood gas and metabolite dynamics, clinical blood and bone marrow, and organ mass and morphology. All methods and results are described in detail in our study so that any researcher can reproduce our fixed-volume HS model for their own scientific purposes.

## 5. Conclusions

We developed a suitable model of HS in male SD rats, mimicking the condition that develops in humans following massive blood loss of various etiologies. This model can be easily reproduced in most preclinical laboratories and used in studies exploring therapeutic approaches to correcting HS.

## Figures and Tables

**Table 1:** Description of groups and procedures.

Group Number	Drug/Code Name in Study	Administration Scheme	Animal Numbers	
			Euthanasia on 8th Day After HS	Euthanasia on 29th Day After HS
1	Saline (NaCl 0.9%) + Albumin 20% (7:1)/SA	HS (blood withdrawal 40% of CBV) + VRT 50% of withdrawn volume	1–5	6–10
2	Autologous blood/AB		11–15	16–20
3	Intact/INT	No impact	21–25	26–30

**Table 2:** Blood sample collection scheme.

Time Point	Volume, µL		
	Hematology	Hemostasis	Gas Analyzer
Before HS	200	500	75
Before VRT (60 min after HS start)	200	-	75
3 h after VRT	200	-	75
24 h after HS	200	500	75
48 h after HS	200	500	75
8 days after HS	200	500	-
29 days after HS	200	500	-

**Table 3:** Parameters measured in blood using ABL90 Flex gas analyzer.

Parameter	Description
pCO_2_ (mmHg)	Partial pressure of carbon dioxide (the respiratory component of acid-base balance, reflecting the adequacy of pulmonary ventilation)
pO_2_ (mmHg)	Partial pressure of oxygen (reflects only a small fraction (1–2%) of the total oxygen in the blood dissolved in the blood plasma). The remaining 98–99% of the oxygen present in the blood is bound to hemoglobin in red blood cells. pO_2_ primarily reflects the absorption of oxygen by the lungs [49].
ctHb (g/dL)	Total hemoglobin concentration is concentration of all forms of hemoglobin in the blood.
cGlu (mmol/L)	Blood glucose concentration (glucose is the most abundant carbohydrate in mammalian metabolism and serves as a major source of intracellular energy)
cLac (mmol/L)	Blood lactate anion concentration (lactate anion is the result of lactic acid dissociation).
cBase (Ecf) (mmol/L)	An indicator of standard excess (or deficit) of bases in all extracellular fluids of the body. It reflects the metabolic component of acid-base imbalances. Positive values indicate an excess of bases (metabolic alkalosis), while negative values indicate a deficit (metabolic acidosis).
CaO_2_ (mL)	Total amount of oxygen transported in arterial blood, combining oxygen bound to hemoglobin and oxygen dissolved in plasma. Arterial oxygen content was calculated as CaO_2_ (mL) = 1.34 × Hb (g/dL) × SO_2_ + 0.0031 × PO_2_ (mmHg) [50,51].

**Table 4:** A scoring scale for assessing severity of pathohistological changes.

Score	Severity	Description
0	Within normal range	The organ tissue appears normal, consistent with the conditions of the study, age, sex, and strain of the animal. Minor deviations may be present, which are considered normal variations.
1	Minimal	Observed change in tissue barely deviates from the normal state.
2	Slight	Tissue damage is easily identified, but the severity is minor.
3	Moderate	Moderate tissue change.
4	Marked	Tissue change is noticeable, but there is potential for increased severity.
5	Severe	The degree of change is maximum (and/or occupies most of the organ).
P	Present	It is set when it is impossible to express a feature on a point scale (only its presence is recorded).

**Table 5:** Body weight gain, %.

	Group 1 SA		Group 2 AB		Group 3 INT	
Day of the Study	Mean ± SD	N	Mean ± SD	N	Mean ± SD	N
Day 7	−9.3 ± 5.0 *** ###	8	−6.9 ± 2.9 *** ###	10	9.1 ± 1.7	10
Day 14	−1.0 ± 5.1 *** ###	4	−3.0 ± 2.8 ###	5	18.7 ± 2.0 ***	5
Day 21	1.0 ± 3.8 *** ###	3	0.1 ± 2.7 ###	5	27.7 ± 3.6 ***	5
Day 28	5.3 ± 4.4 ###	3	3.9 ± 2.0 ###	5	35.3 ± 5.3 ***	5

*** *p* ≤ 0.001 relative to baseline values on day 1, ### *p* ≤ 0.001 relative to intact group (in the figure, statistically significant differences are indicated by brackets) according to Repeated measures ANOVA, post-hoc Fisher LSD test.

**Table 6:** Dynamics of changes in hematological parameters.

	BL		HS Before VRT		3 h After HS		24 h After HS		48 h After HS		8 Days After HS		29 Days After HS	
	SA	AB	SA	AB	SA	AB	SA	AB	SA	AB	SA	AB	SA	AB
	N = 10	N = 10	N = 10	N = 10	N = 8	N = 10	N = 8	N = 10	N = 8	N = 10	N = 4	N = 5	N = 3	N = 5
WBC, 10^9^/L	5.7 ± 1.4	5.2 ± 0.5	6.4 ± 0.8	6.9 ± 0.8	9.0 ± 1.6	** ↑ 9.5 ± 1.2	*** ↑ 11.6 ± 3.3	* ↑ @ ↓ 10.7 ± 3.2	*** ↑ 15.3 ± 5.4	**@ ↓** 8.7 ± 2.9	*** ↑ 15.3 ± 5.5	** ↑ @ ↓ 9.4 ± 2.3	6.9 ± 0.8	7.4 ± 1.2
LYM, 10^9^/L	4.1 ± 1.1	3.8 ± 0.5	4.7 ± 0.6	5.0 ± 0.8	* ↓ 2.3 ± 0.4	* ↓ 2.4 ± 0.8	5.9 ± 1.8	6.2 ± 2.4	*** ↑ 10.4 ± 3.3	**@ ↓** 5.9 ± 1.9	*** ↑ 10.8 ± 3.6	** ↑ 6.4 ± 1.6	5.0 ± 0.7	* ↑ @ ↑ 5.7 ± 1.2
MON, 10^9^/L	0.5 ± 0.2	0.4 ± 0.2	0.8 ± 0.4	0.9 ± 0.5	* ↑ 1.3 ± 0.7	**@ ↓** 0.9 ± 0.5	** ↑ 1.5 ± 0.8	**@@ ↓** 1.3 ± 1.0	1.1 ± 0.6	**@ ↓** 0.7 ± 0.6	*** ↑ 2.3 ± 2.0	**@@@ ↓** 0.8 ± 0.2	0.5 ± 0.2	0.4 ± 0.1
GRA, 10^9^/L	1.0 ± 0.4	1.0 ± 0.4	1.0 ± 0.5	1.1 ± 0.6	** ↑ 5.3 ± 1.3	*** ↑ 6.2 ± 1.1	*** ↑ 4.2 ± 1.8	** ↑ 3.2 ± 1.7↑	*** ↑ 3.8 ± 2.5	**@ ↓** 2.1 ± 0.7	2.3 ± 1.1	2.3 ± 1.2	1.3 ± 0.2	1.3 ± 0.3
LYM, %	73.8 ± 4.2	73.7 ± 5.8	73.7 ± 4.0	71.2 ± 5.5	*** ↓ 26.5 ± 4.3	*** ↓ 25.5 ± 6.8	*** ↓ 52.1 ± 11.6	*** ↓ 57.4 ± 16.2	70.1 ± 12.7	70.6 ± 5.0	71.8 ± 11.3	70.8 ± 5.9	72.8 ± 1.8	76.4 ± 6.2
MON, %	9.4 ± 3.4	7.5 ± 3.0	11.4 ± 5.6	12.2 ± 6.3	*** ↑ 14.7 ± 7.4	**@ ↓** 9.6 ± 5.4	11.8 ± 4.7	11.3 ± 5.7	7.0 ± 2.1	7.1 ± 3.7	*** ↑ 13.0 ± 8.5	**@ ↓** 8.0 ± 0.9	7.7 ± 1.4	6.0 ± 0.8
GRA, %	16.8 ± 4.8	18.7 ± 7.4	15.0 ± 7.7	16.6 ± 10.5	*** ↑ 58.8 ± 7.4	*** ↑ 64.9 ± 11.0	*** ↑ 36.2 ± 11.0	*** ↑ 31.2 ± 16.8	22.9 ± 11.3	22.4 ± 4.5	15.3 ± 4.5	21.2 ± 5.9	19.6 ± 2.8	17.6 ± 6.0
RBC, 10^12^/L	8.4 ± 0.3	8.0 ± 0.2	*** ↓ 5.3 ± 0.6	*** ↓ 5.3 ± 0.3	*** ↓ 4.6 ± 0.7	*** ↓ @@ ↑ 6.1 ± 0.4	*** ↓ 4.5 ± 0.2	*** ↓ @@ ↑ 6.2 ± 0.5	*** ↓ 4.2 ± 0.4	*** ↓ @@ ↑ 5.7 ± 0.8	*** ↓ 5.6 ± 0.2	*** ↓ @ ↑ 6.4 ± 0.2	*** ↓ 7.1 ± 0.5	* ↓ 7.6 ± 0.3
HGB, g/L	147 ± 4	143 ± 4	*** ↓ 95 ± 8	*** ↓ 93 ± 5	*** ↓ 81 ± 12	*** ↓ @@ ↑ 107 ± 7	*** ↓ 80 ± 4	*** ↓ @@ ↑ 111 ± 10	*** ↓ 78 ± 8	*** ↓ @@ ↑ 104 ± 14	*** ↓ 121 ± 6	*** ↓ 128 ± 1	*** ↓ 141 ± 6	147 ± 5
HCT, L/L	0.51 ± 0.02	0.49 ± 0.04	*** ↓ 0.33 ± 0.03	*** ↓ 0.33 ± 0.02	*** ↓ 0.28 ± 0.04	*** ↓ @@@ ↑ 0.37 ± 0.03	*** ↓ 0.28 ± 0.01	*** ↓ @@@ ↑ 0.38 ± 0.03	*** ↓ 0.27 ± 0.02	*** ↓ @@@ ↑ 0.35 ± 0.04	*** ↓ 0.44 ± 0.02	*** ↓ 0.43 ± 0.01	0.47 ± 0.02	0.49 ± 0.02
MCV, fL	60.2 ± 1.7	61.0 ± 0.9	60.8 ± 1.9	61.6 ± 1.2	60.6 ± 1.6	61.4 ± 1.0	61.1 ± 1.1	61.5 ± 1.2	** ↑ 64.2 ± 1.5	62.5 ± 1.6	*** ↑ 77.2 ± 4.4	*** ↑ @@ 66.5 ± 2.8	*** ↑ 66.9 ± 2.7	*** ↑ 64.3 ± 1.3
MCH, pg	17.6 ± 0.5	17.9 ± 0.4	17.5 ± 0.7	17.7 ± 0.3	17.5 ± 0.7	17.7 ± 0.3	17.7 ± 0.4	17.9 ± 0.3	** ↑ 17.6 ± 2.2	18.4 ± 0.4	*** ↑ 21.5 ± 0.3	*** ↑ @ ↓ 19.9 ± 0.6	*** ↑ 20.0 ± 0.5	*** ↑ 19.4 ± 0.1
MCHC, g/L	292 ± 8	293 ± 7	288 ± 10	287 ± 6	289 ± 8	288 ± 7	289 ± 5	292 ± 10	287 ± 9	294 ± 8	*** ↓ 279 ± 16	**@@ ↑** 299 ± 6	299 ± 6	302 ± 5
RDW, %	11.9 ± 0.5	11.7 ± 0.4	11.6 ± 0.5	11.8 ± 0.4	11.6 ± 0.4	11.9 ± 0.4	11.6 ± 0.4	11.8 ± 0.5	10.9 ± 0.6	11.5 ± 0.6	*** ↑ 18.7 ± 1.6	*** ↑ @@@ ↓ 14.3 ± 1.1	*** ↑ 13.9 ± 0.3	*** ↑ 13.0 ± 0.4
RDW-SD, fL	30.4 ± 1.5	30.9 ± 1.5	30.9 ± 2.0	31.3 ± 1.5	30.3 ± 1.5	30.9 ± 1.0	33.6 ± 1.8	32.3 ± 2.0	33.0 ± 1.9	32.8 ± 1.7	*** ↑ 60.9 ± 10.7	*** ↑ @@@ 40.8 ± 4.4	*** ↑ 39.2 ± 1.2	*** ↑ 36.4 ± 2.5
PLT, 10^9^/L	779 ± 129	765 ± 88	823 ± 170	807 ± 118	640 ± 70	* ↓ 655 ± 79	786 ± 112	* ↓ @ ↓ 663 ± 151	834 ± 144	* ↓ @@ ↓ 634 ± 93	*** ↑ 1763 ± 397	* ↑ @@@ ↓ 1467 ± 121	786 ± 113	* ↑ 787 ± 80
MPV, fL	6.0 ± 0.2	5.8 ± 0.4	5.8 ± 0.3	5.8 ± 0.4	5.6 ± 0.2	5.6 ± 0.2	5.9 ± 0.2	5.8 ± 0.2	5.6 ± 0.1	5.6 ± 0.2	5.3 ± 0.3	*** ↓ 5.1 ± 0.1	5.7 ± 0.4	5.7 ± 0.2
PCT, cL/L	0.5 ± 0.1	0.5 ± 0.0	0.5 ± 0.1	* ↑ 0.7 ± 0.1	0.4 ± 0.0	0.4 ± 0.0	0.5 ± 0.1	0.4 ± 0.1	0.5 ± 0.1	** ↓ @ ↓ 0.4 ± 0.1	*** ↑ 0.9 ± 0.3	*** ↑ @@@ ↓ 0.8 ± 0.1	0.5 ± 0.0	0.5 ± 0.0
PDW, %	16.4 ± 1.7	15.5 ± 1.4	* ↓ 14.7 ± 0.7	* ↓ 14.0 ± 0.9	** ↓ 14.0 ± 1.1	14.1 ± 0.8	* ↓ 14.5 ± 0.8	14.8 ± 1.1	** 13.2 ± 0.3	13.8 ± 0.8	*** ↓ 12.3 ± 0.8	*** ↓ 12.5 ± 0.3	** ↓ 14.1 ± 1.5	* ↓ 14.0 ± 0.4
P-LCR	3.4 ± 1.2	5.2 ± 0.5	2.9 ± 1.3	3.0 ± 1.6	2.4 ± 0.6	2.2 ± 0.8	3.1 ± 0.5	** ↓ 2.8 ± 0.8	2.6 ± 0.3	2.7 ± 0.9	1.7 ± 0.6	** ↓ 1.1 ± 0.2	2.2 ± 0.8	2.2 ± 0.6

* *p* ≤ 0.05, ** *p* ≤ 0.01,*** *p* ≤ 0.001 relative to BL, @ *p* ≤ 0.05, @@ *p* ≤ 0.01, @@@ *p* ≤ 0.001 relative to the SA group at the corresponding time point according to Repeated measures ANOVA with post-hoc Fisher LSD test. The arrow next to the significance icon indicates the vector of parameter change (↑ increase, ↓ decrease).

**Table 7:** Organ weight on days 8 and 29 after HS.

	Group 1 SA		Group 2 AB		Group 3 INT	
	Day 8 N = 4	Day 29 N = 3	Day 8 N = 5	Day 29 N = 5	Day 8 N = 5	Day 29 N = 5
Body weight, g	305 ± 18	352 ± 7	301 ± 31	347 ± 15	304 ± 18	349 ± 19
**Absolute organ weight, g**						
Spleen	1.17 ± 0.07 *** ↑	0.97± 0.04 ### ↓	1.00 ± 0.16 ** ↑ @ ↓	0.92 ± 0.13	0.79 ± 0.08	0.80 ± 0.08
Adrenal glands	0.049 ± 0.006	0.045 ± 0.001 * ↓	0.050 ± 0.005	0.048 ± 0.002	0.048 ± 0.006	0.054 ± 0.006
Kidneys	1.76 ± 0.06 *** ↓	1.88 ± 0.09 *** ↓	1.89 ± 0.22 *** ↓	1.91 ± 0.19 *** ↓	2.42 ± 0.22	2.59 ± 0.18
Liver	10.5 ± 0.6 ** ↓	12.4 ± 0.3 # ↑	10.5 ± 1.5 ** ↓	11.7 ± 0.7	12.3 ± 1.0	12.6 ± 1.3
Submandibular lymph nodes	0.033 ± 0.005	0.037 ± 0.006	0.025 ± 0.006	0.034 ± 0.010	0.037 ± 0.010	0.036 ± 0.011
Thymus	0.11 ± 0.02 *** ↓	0.25 ± 0.03 *** ↓ ## ↑	0.16 ± 0.04 *** ↓	0.26 ± 0.09 *** ↓	0.58 ± 0.06	0.60 ± 0.08
Lungs	1.7 ± 0.1	1.8 ± 0.1	1.7 ± 0.2	1.7 ± 0.1	1.9 ± 0.5	1.8 ± 0.3
Brain	1.7 ± 0.1 * ↓	1.8 ± 0.1 ** ↓	1.7 ± 0.1 * ↓	1.8 ± 0.1	1.9 ± 0.1	1.9 ± 0.07
**Relative organ weight (relative to body weight at necropsy), %**						
Spleen	0.38 ± 0.02 *** ↑	0.27 ± 0.01 ### ↓	0.33 ± 0.04 ** ↑ @ ↑	0.27 ± 0.04 ## ↓	0.26 ± 0.03	0.23 ± 0.03
Adrenal glands	0.016 ± 0.002	0.013 ± 0.001 # ↓	0.017 ± 0.001	0.014 ± 0.001 # ↓	0.016 ± 0.002	0.016 ± 0.002
Kidneys	0.58 ± 0.04 *** ↓	0.53 ± 0.02 *** ↓	0.63 ± 0.05 *** ↓	0.55 ± 0.04 *** ↓ # ↓	0.80 ± 0.08	0.74 ± 0.05
Liver	3.4 ± 0.06 *** ↓	3.52 ± 0.14	3.48 ± 0.20 *** ↓	3.37 ± 0.15	4.05 ± 0.23	3.61 ± 0.27
Submandibular lymph nodes	0.011 ± 0.002	0.010 ± 0.002	0.008 ± 0.001	0.010 ± 0.003	0.012 ± 0.004	0.010 ± 0.004
Thymus	0.036 ± 0.008 *** ↓	0.072 ± 0.009 *** ↓ # ↑	0.054 ± 0.0146 *** ↓	0.074 ± 0.030 *** ↓	0.190 ± 0.017	0.172 ± 0.021
Lungs	0.558 ± 0.027	0.504 ± 0.012	0.546 ± 0.026	0.494 ± 0.021	0.618 ± 0.148	0.505 ± 0.110
Brain	0.562 ± 0.020	0.506 ± 0.020	0.564 ± 0.040	0.530 ± 0.049	0.611 ± 0.038	0.551 ± 0.025

* *p* ≤ 0.05, ** *p* ≤ 0.01, *** *p* ≤ 0.001 relative to intact group on the corresponding day of necropsy, # *p* ≤ 0.05, ## *p* ≤ 0.01, ### *p* ≤ 0.001 relative to day 8 within the group, @ *p* ≤ 0.05 relative to the SA group on the corresponding day of necropsy according to one-way ANOVA, post-hoc Fisher LSD test. The arrow next to the significance icon indicates the vector of parameter change (↑ increase, ↓ decrease). Data are presented as mean ± standard deviation.

**Table 8:** Histological changes on the 8th day after HS.

Group	SA	AB
**Spleen (number of samples)**	**4**	**5**
**White pulp cell hyperplasia** (individual score 0–5 (***average score***))	3/3/3/3 (***3.0 ± 0.0***)	3/3/2/3/3 (***2.8 ± 0.4***)
**Increased extramedullary hematopoiesis in red pulp** (individual score 0–5 (***average score***))	3/3/3/3 (***3.0 ± 0.0***)	3/3/3/3/3 (***3.0 ± 0.0***)
**Kidneys (number of samples)**	**4**	**5**
**Acute focal ischemia** (present “+”, absent “−” (***“+” percentage, %***))	+/−/+/− (***50.0%***)	+/−/+/+/+ (***80.0%***)
**Thymus (number of samples)**	**4**	**5**
**Accidental involution** (individual score 0–5 (***average score***))	4/3/3/3 (***3.3 ± 0.5***)	2/3/0/2/2 (***1.8 ± 1.1***) *
**Decreased corticomedullary ratio** (individual score 0–5 (***average score***))	4/4/4/4 (***4.0 ± 0.0***)	3/4/2/3/3 (***3.0 ± 0.7***) *
**Brain (number of samples)**	**4**	**5**
**Neuronal death in projection of CA1, CA2, CA3 and dentate gyrus of hippocampus** (number of cases (***“+” percentage, %***))	1 (***25.0%***)	1 (***20.0%***)
**Foci of necrosis in projection of basal ganglia** (number of cases (***“+” percentage, %***))	1 (***25.0%***)	0 (***0.0%***)

* *p* ≤ 0.05 relative to the SA group according to the Mann-Whitney U-test for pairwise comparison. Average scores are presented as mean values ± standard deviation.

**Table 9:** Histological changes on the 29th day after HS.

Group	SA	AB
**Spleen (number of samples)**	**3**	**5**
**White pulp cell hyperplasia** (individual score 0–5 (***average score***))	2/2/2 (***2.0 ± 0.0***)	2/2/2/2/2 (***2.0 ± 0.0***)
**Kidneys (number of samples)**	**3**	**5**
**Focal post-ischemic nephrosclerosis** (present “+”, absent “−” (***“+” percentage, %***))	−/−/+ (***33.3%***)	−/+/+/−/− (***40.0%***)
**Thymus (number of samples)**	**3**	**5**
**Decreased corticomedullary ratio** (individual score 0–5 (***average score***))	2/2/2 (***2.0 ± 0.0***)	2/2/2/2/2 (***2.0 ± 0.0***)
**Brain (number of samples)**	**3**	**5**
**Gliomesodermal scars in projection of parietal cortex** (number of cases (***“+” percentage, %***))	1 (***33.3%***)	0 (***0.0%***)
**Gliomesodermal scars in cerebellar cortex** (number of cases (***“+” percentage, %***))	1 (***33.3%***)	0 (***0.0%***)

## Data Availability

The original contributions presented in this study are included in the article. Further inquiries can be directed to the corresponding author.

## Abbreviations

The following abbreviations are used in this manuscript:

HS	hemorrhagic shock
CBV	circulating blood volume
AP	arterial pressure
HR	heart rate
TF	transfusion
VRT	volume resuscitation therapy
AB	autologous blood
AS	albumin-containing saline (NaCl 0.9% + Albumin 20% (7:1))
INT	intact
SD rat	Sprague Dawley rat
SPF	specific pathogen free
cBase (Ecf)	calculated Base, Extracellular fluid
pCO_2_	carbon dioxide partial pressure
pO_2_	oxygen partial pressure
ctHb	total hemoglobin concentration
cGlu	total glucose concentration
cLac	total lactate concentration
CaO_2_	total amount of oxygen transported in arterial blood
APTT	activated partial thromboplastin time
PT	prothrombin time
Fg	fibrinogen
K_3_EDTA	tripotassium edetate
RBC	red blood cell
Hb	hemoglobin
HCT	hematocrit
WBC	white blood cell
MCH	mean corpuscular haemoglobin
MCHC	mean cell haemoglobin concentration
MCV	mean corpuscular volume
RDW	red cell distribution width—variation coefficient
RDW-SD	red cell distribution width-standard deviation
PLT	platelets
MPV	mean platelet volume—variation coefficient
PCT	plateletcrit
PDW	platelet distribution width—variation coefficient
LYM	lymphocytes
MON	monocytes
GRA	granulocytes
PBS	phosphate-buffer saline
HPCs	hematopoietic progenitor cells
SIRS	systemic inflammatory response syndrome
IL	interleukin
ATP	adenosine triphosphate
IFN	interferon
Mip	macrophage inflammatory protein
NF	nuclear factor
